# Metal Nanoparticle-Flavonoid Connections: Synthesis, Physicochemical and Biological Properties, as Well as Potential Applications in Medicine

**DOI:** 10.3390/nano13091531

**Published:** 2023-05-02

**Authors:** Stepan Sysak, Beata Czarczynska-Goslinska, Piotr Szyk, Tomasz Koczorowski, Dariusz T. Mlynarczyk, Wojciech Szczolko, Roman Lesyk, Tomasz Goslinski

**Affiliations:** 1 Chair and Department of Chemical Technology of Drugs, Poznan University of Medical Sciences, Grunwaldzka 6, 60-780 Poznań, Poland; 2 Doctoral School, Poznan University of Medical Sciences, Bukowska 70, 60-812 Poznań, Poland; 3 Chair and Department of Pharmaceutical Technology, Poznan University of Medical Sciences, Grunwaldzka 6, 60-780 Poznań, Poland; 4 Department of Biotechnology and Cell Biology, Medical College, University of Information Technology and Management in Rzeszów, Sucharskiego 2, 35-225 Rzeszow, Poland; 5 Department of Pharmaceutical, Organic and Bioorganic Chemistry, Danylo Halytsky Lviv National Medical University, Pekarska 69, 79010 Lviv, Ukraine

**Keywords:** cancer, flavonoids, metal nanoparticles, polyphenols, reactive oxygen species

## Abstract

Flavonoids are polyphenolic compounds widely occurring throughout the plant kingdom. They are biologically active and have many medical applications. Flavonoids reveal chemopreventive, anticarcinogenic, and antioxidant properties, as well as being able to modulate the immune system response and inhibit inflammation, angiogenesis, and metastasis. Polyphenols are also believed to reverse multidrug resistance via various mechanisms, induce apoptosis, and activate cell death signals in tumor cells by modulating cell signaling pathways. The main limitation to the broader usage of flavonoids is their low solubility, poor absorption, and rapid metabolism. To tackle this, the combining of flavonoids with nanocarriers could improve their bioavailability and create systems of wider functionalities. Recently, interest in hybrid materials based on combinations of metal nanoparticles with flavonoids has increased due to their unique physicochemical and biological properties, including improved selectivity toward target sites. In addition, flavonoids have further utilities, even in the initial step of preparation of metal nanomaterials. The review offers knowledge on multiple possibilities of the synthesis of flavonoid-metal nanoparticle conjugates, as well as presents some of their features such as size, shape, surface charge, and stability. The flavonoid-metal nanoparticles are also discussed regarding their biological properties and potential medical applications.

## 1. Introduction

Flavonoids represent a category of polyphenolic compounds found throughout the plant kingdom that has received much interest due to their numerous biological activities [1]. They have chemopreventive, anticarcinogenic properties, and demonstrate antiproliferative activity on tumor cells. In addition, they inhibit inflammation, angiogenesis, and metastasis [2]. Polyphenols are also believed to reverse multidrug resistance via various mechanisms as well as induce apoptosis and activate cell death signals in tumor cells by modulating cell signaling pathways, such as activator protein-1, nuclear factor NF-kappa-B (NF-κB) or mitogen-activated protein kinases [1,3]. Of importance is the modulation of the immune system response by flavonoids as well as their antioxidant activity, which manifests itself through the ability of free radical scavenging [2,4]. Despite their promising health-promoting potential, numerous flavonoids, including (−)-epigallocatechin-3-*O*-gallate (EGCG), quercetin (QUR), genistein, apigenin (AP), naringenin, silibinin, and kaempferol, reveal low solubility, poor absorption, and rapid metabolism. Therefore, combining flavonoids with nanocarriers could improve their bioavailability and create systems of wider functionalities [5].

In recent years, nanoparticles have attracted the broad attention of researchers dealing with various scientific disciplines, mainly due to their interesting physicochemical properties and potential applicability in medicine. Therefore, interest in combining flavonoids with metal nanoparticles (NPs) has increased due to the unique physical, chemical, and biological properties of the resulting connections or hybrid materials. Chemical reduction of the noble metal precursor is one of the most popular methods of preparation of noble metal nanoparticles. Metal nanomaterials formulated with active substances of plant origin can be synthesized using physical, chemical, or biological methods and characterized with analytical techniques involving microscopic and spectroscopic studies. Other benefits of NPs are low cost, simple synthesis, and the possibility of controlling both their shapes and their sizes. It is also interesting that flavonoids themselves are useful for the preparation of metal nanomaterials, with these being reducing and electrostatic agents for the ‘Green’ synthesis of NPs from their metal salt precursors [6,7,8,9]. In metal nanoparticles, features of metals are exploited, such as optical polarizability, electrical conductivity, chemical properties, antibacterial effectiveness, and biocompatibility [10].

Metal nanoparticles are becoming more and more widely considered as perspective pharmaceutical carriers because of their numerous advantages. Metal nanoparticles have been developed as a superior alternative to conventional cancer therapy treatment due to theranostic properties that provide both diagnosis and drug delivery designed to monitor the therapy [11]. Noble metal NPs enable the tracking of nano-complex therapeutic carriers within the body owing to their unique plasmonic properties, which makes such therapy more efficient and safer. Regarding non-noble metal NPs, they are cost-effective, can convert electromagnetic energy into heat (hyperthermia), and possess magnetic properties [12,13]. Metal nanoparticles present increased stability and half-life in circulation, as well as appropriate biodistribution [14]. Due to the surface modification and incorporation of different ligands, they can target specific tissues and cells [4]. Functionalizing the nanoparticles with flavonoid ligands was found to improve their selectivity and allow them to reach the target sites [8].

**Figure 1:**
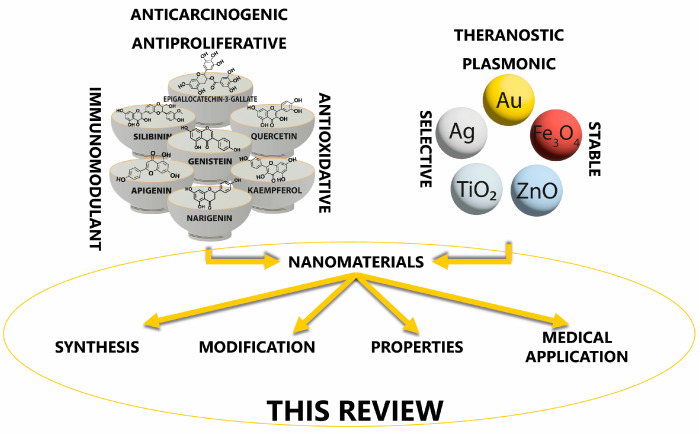
Flavonoid-metal nanoparticle conjugates and hybrids discussed in the review in terms of their synthesis, physicochemical, and biological features.

Overall, this review summarizes the current state of flavonoid-metal nanoparticle conjugates and hybrids, including multiple possibilities for their synthesis and modifying features such as size, shape, surface charge, and stability (Figure 1). The flavonoid-metal nanoparticles are also discussed regarding their biological properties and medical applications, including potential utility as drug delivery vehicles, modulators of cellular responses, applications in cancer therapy, antibacterial treatment, and tissue engineering.

## 2. Physicochemical and Biological Properties of Flavonoids

**Figure 2:**
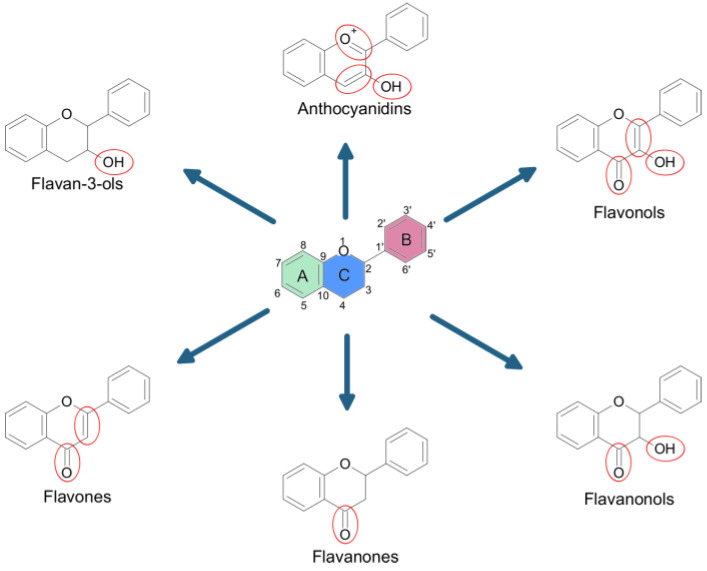
A 2-phenylchromane scaffold of flavonoids with a heterocyclic pyran ring (C) fused with the benzene ring (A) and linked to the phenyl ring (B). Selected subclasses of flavonoids.

Flavonoids are secondary plant metabolites, often acting as pigments responsible for the colors of fruit, flowers, and vegetables. They are characterized by a 2-phenylchromane scaffold (C6-C3-C6) with a heterocyclic pyran ring (C) fused with the benzene ring (A) and linked to the phenyl ring (B) (Figure 2). Flavonoid chemical structures have various substituents, including multiple hydroxyls (-OH), methoxyl (-OCH_3_), and glycoside groups, along with an oxo group at position 4 of the C-ring. Flavonoids can be classified into various subclasses based on the oxidation level, unsaturation and substitution pattern of the C-ring, and the bonding position of the B-ring to C2/C3/C4 carbons of the C-ring. The subclasses of flavonoids include flavones, flavonols, flavanones, flavanonols, flavan-3-ols, isoflavones, neoflavonoids, anthocyanidins, chalcones, dihydrochalcones, and aurones. Flavonoids may exist as aglycones or as their derivatives. The structural features and configurations of flavonoids determine their bioavailability, metabolism, biochemical, and pharmacological activities [15].

Flavonoids exhibit unique physicochemical properties that influence their solubility in various solvents. Several factors affect their solubility in water, including the presence of a double bond in the C-ring and the number of hydroxyl (-OH) substituents in the B-ring. The solubility of flavonoids in 1-octanol is adversely affected by the number of hydroxyl groups, whereas the solubility in water generally increases with a rise in the number of hydroxyl groups. The addition of an -OCH_3_ substituent to the B-ring reduces their solubility in both solvents. Additionally, the linkage between C2 and C3 plays a significant role in determining solubility, as the presence of a double bond results in lower solubility in both water and 1-octanol. An -OH substituent at C3 decreases aqueous solubility but increases 1-octanol solubility when the linkage between C2 and C3 is a double bond. The dissolution of flavonoids in 1-octanol can be either entropy-driven (chrysin, apigenin, kaempferol, morin) or enthalpy-determined. Overall, the solubility in water is a critical factor in the spontaneous transfer of flavonoids from water to 1-octanol [16].

Upon spectroscopic examination, flavonoids display two primary absorbance bands. Band I, in the range of 320–385 nm, results from the B-ring absorption, while Band II, within 250–285 nm, originates from the A-ring absorption. Any changes introduced to the structure cause differences in shifts of absorbance bands, e.g., kaempferol at 367 nm, quercetin at 371 nm, and myricetin at 374 nm. Flavanones, featuring a C-ring without a double bond bear different spectral profiles from other flavonoid subclasses. Typically, the spectra of flavanones exhibit a major Band II peak within the 270–295 nm range (288 nm for naringenin or 285 nm for taxifolin) along with a smaller Band I peak (326–327 nm). When a highly substituted B-ring is present, Band II often appears as a multitude of peaks. Anthocyanins, containing cinnamoyl/benzoyl units, display distinct absorbance properties, exhibiting a bandwidth ranging between 450 and 560 nm and another between 240 and 280 nm, subject to modification by hydroxyl groups connected to the B-ring [17]. Flavonoid compounds are sensitive to elevated temperatures encountered during microwave-assisted (MAE) and ultrasonic-assisted solvent extractions (UAE). These molecules may undergo degradation when subjected to excessive thermal stress. Many factors contribute to this phenomenon, including the quantity and positioning of hydroxyl moieties within their structures and the existence of accompanying substituents such as carbohydrates. The extent to which glycosidic bonds impede flavonoid stability appears relatively inconsequential compared to these other influences. Therefore, careful consideration is needed prior to employing MAE or UAE techniques to prevent undue deterioration of target analytes. Although conventional heating approaches have been shown to provide comparable extraction efficacy, they require significantly lengthier durations per unit of mass processed [18].

The composition and arrangement of hydroxyl groups within the A-, B-, and C-rings contribute to flavonoid antioxidant potential. Hydroxyls on the B-ring, particularly those in the *o*-dihydroxy configuration, increase the stability of the radical formed while enhancing electron delocalization. Pyrogallol groups augment antioxidant capacity, whereas an additional C2–C3 double bond and oxo-functionality in the pyran ring promotes further electron delocalization, leading to improved antioxidant performance. Flavonoids with a single 3-OH group and paired hydroxyls at positions 5 or 8 in the A-ring display favorable antioxidant characteristics. The glycosylation of flavonoids typically reduces antioxidant capacity relative to their aglycone forms. The type, location, and structural features of attached sugars play crucial roles in determining antioxidant efficacy. Modifications such as esterification, acylation, methylation, sulfation, and other substitutions commonly diminish antioxidant capacity, with sensitivity towards alterations appearing more prominent in the B-ring than in other regions. Researchers utilize different experimental methods, each yielding unique results depending on the specific testing conditions employed, so as to better understand flavonoids’ antioxidative capabilities [19].

Flavonoid stability varies significantly when subjected to external stimuli such as heat and light. Studies have demonstrated that flavonoids undergo oxidative transformations upon exposure to these factors. These reactions lead to diverse outcomes regarding the preservation of their antioxidant potential. While some flavonoids, such as rutin and luteolin 7-*O*-glucoside, show reduced antioxidant capacity following heat treatment, others, such as quercetin, display remarkable resilience. Eriodictyol, another representative flavonoid, exhibits enhanced antioxidant activity upon thermal degradation due to the generation of novel compounds. Mesquitol appears to be highly responsive to both heat and light stress, resulting in improved antioxidant activity post-decomposition. Investigations into the impact of environmental conditions on flavonoids’ antioxidant behavior typically involve studying their concentrated forms (model solutions). This approach allows for greater control over experimental variables and facilitates a better understanding of how heat treatment affects flavonoids [20].

Flavonoids, originally utilized for their dyeing and preservation properties, have emerged as promising candidates for medicinal chemistry due to their remarkable chemical diversity and recognized biological properties. Flavonoids exhibit variations in different aspects of their absorption, metabolism, and bioavailability within living systems. In the body, these compounds occur in different forms (commonly found as polar conjugates, glucuronides, and sulfates in plasma) and are attached primarily to serum albumin. Thus, these factors limit their distribution throughout the body, including entry into the central nervous system (CNS). During digestion, flavonoids undergo alterations involving changes such as glucuronidation in the intestine lumen, enterohepatic recirculation, hydroxylation, and dehydrogenation, predominantly in the liver. Some flavonoids also undergo transformations via bacterial action in the colon. Finally, certain flavonoid metabolites display considerable pharmacological activity both in vitro and potentially in vivo, yet further research remains necessary to fully comprehend how these combinations contribute to overall health effects [21]. The intricate structural framework of flavonoids permits them to interact with various biological macromolecules such as proteins [22], DNA [23], RNA [24], receptors [25], and bacterial cell walls [26], exhibiting diverse biological activities. The pharmacological versatility of flavonoids has broadened their potential utility in different fields of medicine, including preventive care and palliative treatment for life-threatening disorders. Consequently, extensive research endeavors have been focused on unraveling the underlying mechanisms of flavonoid action, paving the way for novel therapeutic interventions based on their multifaceted properties.

Flavonoids reveal a range of biological effects, including antioxidant [27,28,29], anti-inflammatory [30], antiviral [31], anticancer [1,32], and neuroprotective activities [33,34]. These properties can be attributed to the specific chemical structure and functional groups present within each flavonoid molecule. For instance, the hydroxyl groups present in flavonoids can donate hydrogen atoms, leading to the scavenging of free radicals and inhibition of oxidative stress. Moreover, flavonoids can modulate several signaling pathways by interacting with cell surface receptors and enzymes, ultimately regulating cell growth, proliferation, and apoptosis. Furthermore, the ability of flavonoids to chelate metal ions can disrupt the formation of reactive oxygen species and prevent cellular damage.

According to recent studies, flavonoids may have characteristics that aid in wound-healing processes, which were thoroughly discussed in an excellent review by Zulkefli et al. [35]. This is due to their ability to reduce inflammation, promote the formation of new blood vessels, facilitate skin cell renewal, and protect against the damage caused by toxic chemicals. Flavonoids can influence wound healing by modulating the expression of specific markers linked with pathways such as Angiopoietin-1/Tie-2 (Ang-1/Tie-2) [36], Focal Adhesion Kinase (FAK)/Src [37], c-Jun N-Terminal Kinase (JNK) [38], Mitogen-Activated Protein Kinase/Extracellular Signal-Regulated Kinase (MAPK/ERK) [39], Nuclear Factor Erythroid 2-Related Factor 2/Antioxidant Response Element (Nrf2/ARE) [40], Nuclear Factor Kappa B (NF-κB) [41], p38 Mitogen-Activated Kinase (MAPK) [38], Phosphatidylinositol 3-Kinase/Protein Kinase B (PI3K/AKT) [39], Transforming Growth Factor-beta (TGF-β) [36], Transforming Growth Factor/Suppressor of Mothers against Decapentaplegic (TGF-β/Smads) [36], and Wnt/β-catenin [42].

In conclusion, the intricate and diverse chemical structure of flavonoids enables them to interact with a wide range of biological targets, showcasing their potential as a vital tool in medicinal chemistry. The elucidation of the mechanisms behind flavonoid activity will aid in developing novel therapeutic interventions, which may prove beneficial in preventing and treating various diseases.

## 3. Nanoparticle-Flavonoid Connections

Many studies present connections of flavonoids with metal nanoparticles, mainly Ag, Au, oxides of Fe, Zn, and Ti, which resulted in materials of interesting physicochemical and biological properties.

### 3.1. Silver Nanoparticles

**Figure 3:**
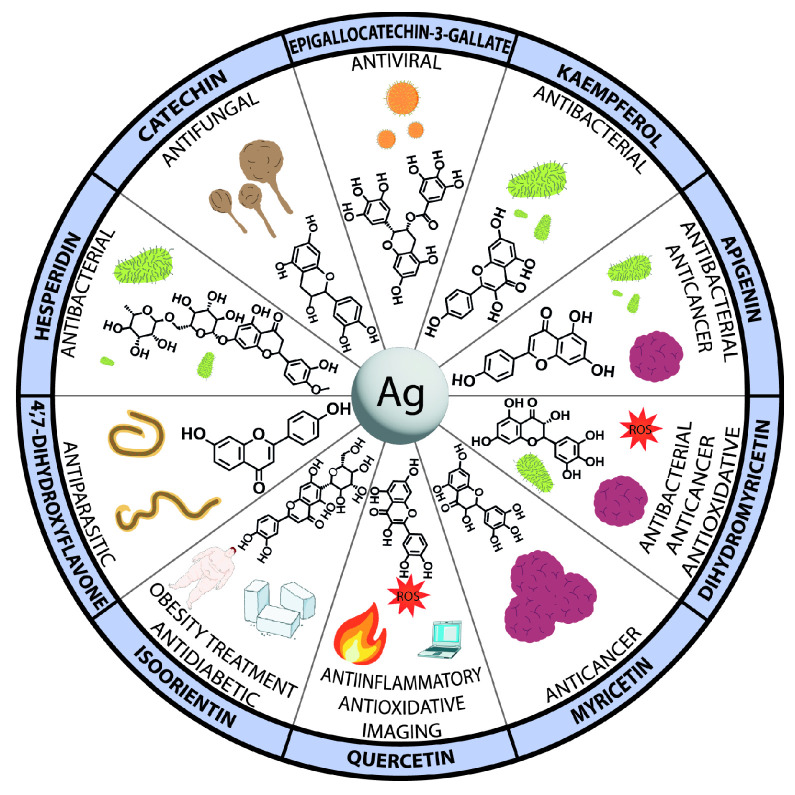
Spectrum of flavonoid-silver nanoparticle conjugates and hybrids with their most striking biological features.

Of great interest are the potential antioxidant, antibacterial, antifungal, antiparasitic, antiviral, and anticancer properties of silver nanoparticles. To study the perspective medical applications, silver nanoparticles were functionalized with apigenin, catechin, EGCG, kaempferol, myricetin, 4′,7-dihydroxyflavone, dihydromyricetin, hesperidin, or quercetin. In addition, silver@quercetin nanoparticles were researched as a biocompatible and photostable aggregation-induced emission luminogen for in situ and real-time monitoring of biomolecules and biological processes. In the biological study, silver nanoparticles with curcumin and quercetin were analyzed in terms of the potential anti-inflammatory effect. Additionally, isoorientin-loaded silver nanoparticles were investigated in terms of their potential toxicity and activity on enzymes related to type II diabetes and obesity (Figure 3).

#### 3.1.1. Silver Nanoparticles in Therapy of Infectious Diseases

**Figure 4:**
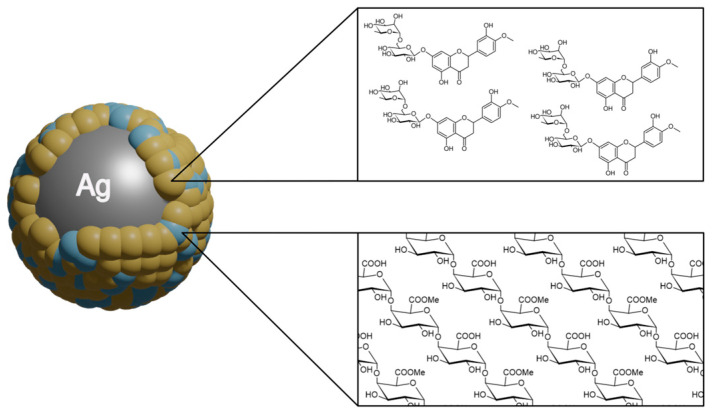
Schematic representation of silver nanoparticles coated with hesperidin and pectin (HP-AgNPs) developed by Zhao et al. [43].

In 2022, Zhao et al. published a study on the synthesis of highly bactericidal silver nanoparticles coated with different ratios of hesperidin and pectin (HP-AgNPs) through a microwave-assisted process (Figure 4) [43]. The prepared AgNPs of 11.93–17.34 nm in size were named according to the ratio of hesperidin to pectin used in the synthesis—P-AgNPs for 0:1, HP-AgNPs1 for 1:3, HP-AgNPs2 for 3:1, and H-AgNPs for 1:0. The proportion of both ingredients revealed a tremendous effect on the morphology of the nanomaterials, as AgNPs prepared with either hesperidin or pectin alone had irregular shapes, while those made with both showed uniform morphology. HP-AgNPs2 demonstrated the most potent antibacterial activity, with a minimum inhibitory concentration (MIC) at 66.7 μg/mL against *Escherichia coli*. This value was significantly lower than the MIC of P-AgNPs, which reached around 8 times higher values at 533.3 μg/mL. The MICs of HP-AgNPs1 and H-AgNPs were also lower than that noted for P-AgNPs, at approximately 266.7 μg/mL and 133.3 μg/mL, respectively. In the study, the combination of hesperidin and pectin with AgNPs significantly enhanced the antimicrobial activity of the nanoparticles against *E. coli*. The adsorbance of HP-AgNPs2 on the cell wall caused significant morphological changes, including depression and damage to their cell wall, which induced oxidative stress and led to bacterial death. The direct contact with bacteria, AgNPs caused cell damage and cytotoxicity related to the release of Ag^+^ ions. The fact that the MIC of HP-AgNPs2 was much lower than that of P-AgNPs, despite the latter releasing 25% more Ag^+^ after 48 h, hints at the possibility of the combined action of particles and Ag^+^ ions contributing to the enhanced antibacterial effect of HP-AgNPs2. Among the AgNPs tested, HP-AgNPs2 showed the highest increase in the amount of reactive oxygen species (up to 262.6%), indicating enhanced antibacterial properties.

In a similar study, ultra-uniform and colloidally stable hesperidin-capped silver nanoparticles (Ag-Hes NPs) were explored in terms of potential use for the treatment of infected wounds [44]. To prepare Ag-Hes NPs, AgNO_3_, and hesperidin solutions were mixed with silver nanoparticles and stirred at room temperature. The resulting Ag-Hes NPs were combined with poly(vinyl alcohol)-sodium alginate (PVA-Alg) and electrospun to form Ag-Hes@H nano hydrogels. A variety of techniques was used to characterize their physicochemical properties, as well as to observe the expression of related proteins in cells and detect apoptosis-related proteins. The Ag-Hes NPs were highly uniform and colloidally stable, with a diameter of around 20 nm and a core-shell structure comprising a hesperidin shell surrounding a Ag core. The antibacterial activity evaluation showed that Ag-Hes NPs reveal a higher reduction in turbidity of *S. aureus* suspension compared to bare Ag nanoparticles. The viability of *S. aureus* decreased significantly in the presence of both Ag and Ag-Hes NPs, with a higher inhibition rate of 94.5% for Ag-Hes compared to 62% for Ag NPs. This may be ascribed to the uniform size and antioxidant activity of Ag-Hes NPs, which was confirmed experimentally. Unlike AgNPs, Ag-Hes NPs were found to be non-toxic as evaluated on human umbilical vein endothelial cells (HUVECs), possibly due to the protective effect of the hesperidin shell. As mentioned, Ag-Hes NPs were used to prepare electro-spun nanofibers and a hydrogel (Ag-Hes@H). The latter was found to significantly enhance the migration of HUVECs cells in a cell scratch assay, as shown by the significant reduction in the size of the scratch gap and the higher migration rate compared to control and other treatment groups. Moreover, Ag-Hes@H improved the closure rate of infected wounds in male rats (97% for the Ag-Hes@H group, and 83% for the Ag@H group after treatment). The most complete process of re-epithelialization and the strongest collagen fibers in the wound healing process of rats, as well as an increase in collagen deposition and proliferation of skin cells at the wound surface, were observed for the Ag-Hes@H group. In further in-depth tests, an increased expression of bFGF (basic fibroblast growth factor, a protein involved in skin regeneration) and SIRT1 was noted, while the expression of NF-κB was decreased. Additionally, the levels of the inflammatory factors MMP9, TNF-α, and IL-6 were suppressed in the Ag-Hes@H group. The breakdown of the gel network and the release of silver core and hesperidin molecules upon exposure to reducing substances in the body may contribute to the inhibitory effects on bacterial growth and inflammation observed with Ag-Hes@H.

In their study from 2021, Kannanoor et al. investigated silver nanoparticles (AgNPs) conjugated with kaempferol and hydrocortisone to formulate KH-AgNPs, which showed strong antibacterial properties against various bacterial strains [45]. KH-AgNPs were synthesized by mixing kaempferol, hydrocortisone, and AgNO_3_ solutions with subsequent heating. Next, the mixture was enriched with NaBH_4_, alkalized with NaOH to pH 12, stirred, cooled, and centrifuged, yielding uniform KH-AgNPs with a diameter of 10–30 nm and a face-centered cubic crystal structure. KH-AgNPs decreased the bacterial growth of *E. coli* with an MIC of 62.5 μg/mL and an MBC of 125 μg/mL. The results of the ROS assay showed that KH-AgNPs dramatically increased oxidative stress in *E. coli* in a concentration-dependent manner, with all KH-AgNP-treated cells producing more ROS than untreated cells. The authors also investigated the effect of KH-AgNPs on *E. coli* biofilms and discovered that KH-AgNPs effectively reduced and dissolved the biofilms by decreasing the colonization of bacteria on the surface. In a separate test, the effect of KH-AgNPs on static biofilm formation was tested at different concentrations. It turned out that treatment with KH-AgNPs for 48 h resulted in a 58% decrease in biofilm formation at a concentration of 110 μg/mL. Treatment with KH-AgNPs led to an increase in the production of free radicals in *E. coli* cells, resulting in lipid peroxidation, which compromises the integrity of the bacterial cell membrane.

Scroccarello et al. recently evaluated the antifungal effectiveness of silver nanoparticles made with several types of PCs with variable antioxidant capabilities, including flavonoids, such as CT and MY [46]. The AgNPs were synthesized by rapid and simple mixing of AgNO_3_ with PCs in an alkaline environment at room temperature; along with CT and MY, caffeic (CF) and gallic (GA) acids were employed. AgNPs@PCs were characterized by UV-vis spectroscopy, DLS, and TEM. The 2,2′-azinobis-(3-ethylbenzothiazoline-6-sulfonic acid) (ABTS) assay results revealed that PCs had greater antioxidant activity than AgNPs@PCs, CT was more active than MY, and dihydroxylic PCs were more active than tri-hydroxylic PCs. The same pattern was observed when AgNPs were generated with relative PCs, demonstrating that the PCs were preserved in the AgNPs shell even after purification. The Folin–Ciocalteu reagent did not react with all of the AgNPs@PCs, indicating that the phenols were bound to the AgNPs and that free PCs were not present. The effect of AgNPs@PCs on *Aspergillus niger* spore germination was investigated on the separate isotropic growth and vegetative tube germination phases. AgNPs@CT at the concentration of 30 mg/L inhibited isotropic growth in the most significant way, reaching 33%, followed by AgNPs@GA and AgNPs@CF with 24% and 23%, respectively, and AgNPs@MY with 17%. At the same concentration, AgNPs@CT revealed the most significant inhibitory impact on vegetative tube germination at 36%, followed by AgNPs@MY at 25%, AgNPs@GA at 20%, and AgNPs@CF at 15%. The effect of AgNPs@PCs on the vegetative growth of *A. niger* was studied in relation to the mycelial growth inhibition induced by applying AgNPs@PCs at five concentrations (5–60 mg/L) after 10 days of incubation. All AgNPs@PCs inhibited mycelial growth significantly, depending on the type of PCs employed in the synthesis and the concentration evaluated, while free PCs did not influence mycelial development. AgNPs@PCs inhibitory activity followed the antioxidant activity trend: AgNPs@CT > AgNPs@CF > AgNPs@MY > AgNPs@GA. The inhibition of hyphal growth and cell damage/viability was used to assess the efficacy of AgNPs@PCs on the growth of *A. niger*, and the effects on *A. niger* mycelium were studied using SEM and confocal imaging. AgNPs@PCs attached to and clad around hyphae caused a decrease in length and quantity as well as an aberrant growth pattern indicative of cell death. Cell viability experiments revealed that AgNPs@PCs led to significant cell death.

In 2021, Saadh and Aldalaen investigated the potential use of EGCG in combination with silver nanoparticles (AgNPs) and in co-administration with zinc(II) ions as a novel topical therapeutic with multiple effects against H5N1 influenza [47]. AgNPs were conjugated with EGCG by mixing various concentrations of EGCG solution with an aqueous solution of AgNO_3_, followed by the addition of NaBH_4_. The optimal inhibitory concentration of EGCG, both free and conjugated, was deduced to be 60 μM, whereas for zinc sulfate, the optimal concentration concluded 1.5 mg/mL. The combination of EGCG and zinc sulfate showed considerable antiviral activity against the H5N1 virus in embryonated SPF eggs, reducing the logEID_50_/mL value 4.2–5.6 times. Similarly, the combination of EGCG-AgNPs and zinc sulfate demonstrated exceptional antiviral efficacy, with a 7.1 reduction in the logEID_50_/mL value compared with the control group. The MTS proliferation assay with the use of different combinations of EGCG, zinc sulfate, and EGCG-AgNPs did not reveal any significant difference between the applied formulations and the control after 72 h. As a result, at the doses utilized in the study, none of the materials were determined to be cytotoxic.

The potential for the antileishmanial activity of 47DHF-functionalized gold and silver nanoparticles (Au-47DHF and Ag-47DHF) was studied by Sasidharan and Saudagar [48]. Nanoparticles Ag-47DHF were synthesized by adding 47DHF in varying ratios to solutions of AgNO_3_ and NaOH. They ranged in size from 10 to 30 nm, with an average size of 25.1 nm, and exhibited a zeta potential of 40 mV. The Ag-47DHF nanoparticles had drug loading (DL) efficiencies of 64.13% and revealed cumulative drug release of 14.55% at pH 7.4 and 51.29% at pH 5.8, with a burst in the first 10 h, followed by a slow release. Ag-47DHF was more effective against axenic amastigotes than promastigotes, with the IC_50_ values against promastigotes at 0.8483 µg/mL, and against axenic amastigotes at 0.262 µg/mL. A 50% cytotoxic concentration (CC_50_) value at 4.95 µg/mL of Ag-47DHF was noted in macrophages. Ag-47DHF with IC_50_ values at 0.215 µg/mL demonstrated antileishmanial action and targeted intracellular amastigotes. In addition, the infectivity index of 30 and the selectivity index (SI) of 24 were noted for Ag-47DHF. Compared to the infected control, the nanoparticle-administered macrophages generated reduced nitrite concentrations, indicating a decrease in parasite burden. The quantity of ROS (the key trigger of apoptosis induction for 47DHF) formed by Ag-47DHF nanoparticles was higher than that in control H_2_O_2_.

#### 3.1.2. Silver Nanoparticles in Anticancer Therapy

Zarei et al. compared the biological potential of sodium citrate-based (SC-SNPs) and AP-based (AP-SNPs) silver nanoparticles in vitro and in vivo [49]. Either sodium citrate (SC) or AP was dissolved in water, filtered, and added to a AgNO_3_ solution to create corresponding SNP colloids. The nanoparticles were subjected to UV-vis spectroscopy, field emission scanning electron microscopy (FESEM), and particle size analysis, which revealed for both types of nanoparticles homogeneous dispersions, stable zeta potentials, and pseudospherical forms. SNPs were tested for anticancer effects against the MCF-7 breast cancer cell line. Both nanoparticles suppressed MCF-7 cell growth in a dose-dependent manner, although the pro-apoptotic activity of AP-SNPs was the most significant. The morphological alterations seen in cancer cells treated with nanoparticles included cell shrinkage, plasma membrane blebbing, detachment, and destruction. Caspase-3, a fundamental apoptotic-related biomarker, was up-regulated 3.17-fold with AP-SNPs and 1.75-fold with SC-SNPs treatment of MCF-7 when compared to the untreated reference. In the research on mice, SC-SNPs substantially elevated liver enzymes alkaline phosphatase, aspartate transaminase, and alanine transaminase at 50 mg/kg/day, whereas AP-SNPs also significantly increased the same enzymes at 100 mg/kg/day. AP-SNPs cytoprotected mouse hepatocytes and dramatically decreased lipid peroxidation in the mouse liver. In addition, as compared to SC-SNPs, they boosted the expression of the antioxidant enzymes SOD and GPx in the liver of mice. The histopathology results revealed no significant cellular damage in the livers of mice treated for 30 days with either kind of nanoparticle.

Anwer et al. explored silver nanoparticles labeled with myricetin as a possible treatment for colorectal cancer [50]. The synthesis was carried out via microwave assisted (AgNPs-mw) or aging (AgNPs-aging) processes. The AgNPs-mw revealed a higher maximum absorbance than AgNPs-aging, indicating that the bioreduction process was faster for AgNPs-mw. TEM results showed that the synthesized nanoparticles had a spherical shape with a size range of 12–20 nm and a crystallite size of approximately 18 and 17 nm for AgNPs-mw and AgNPs-aging, respectively. The mean particle size, as determined by DLS analysis, was approximately 61 nm. The functional groups of the nanoparticles were identified using FTIR spectra, which showed shifts in the peaks for O-H, C-O, and NAH vibrations, indicating reduction resulting from the synthesis process. Myricetin was found to be effective in decreasing the viability of human colorectal cancer cells (HCT116) in a dose-dependent manner when tested in a cell viability assay with the IC_50_ value for myricetin at 106.87 μg/mL, while the IC_50_ value for silver nanoparticles labeled with myricetin (mAgNPs) was 34.04 μg/mL. mAgNPs showed some cytotoxicity towards HCT116 cells at concentrations below 200 μg/mL, although they were biocompatible with normal cells (HEK-293) up to a concentration of 400 μg/mL. There was no significant effect on the viability of healthy cells when treated with myricetin or mAgNPs. The researchers also conducted gene expression and pathway enrichment analysis using a colorectal cancer gene expression dataset and found several genes differentially expressed in healthy versus primary adenocarcinoma cells, healthy versus adjacent cells, and adjacent versus primary adenocarcinoma cells. They also noted that certain pathways were activated in each of these comparisons.

#### 3.1.3. Silver Nanoparticles in Multimodal Action Materials

In 2021, Li et al. synthesized and characterized silver nanoparticles (AgNPs) functionalized by DMY with strong antioxidant, antibacterial, and anticancer properties, making them potential candidates for use as antimicrobial materials in the food and pharmaceutical industries [51]. To formulate the desired nanoparticles, the solutions of DMY and AgNO_3_ were mixed and heated, leading to a grass-green suspension with black DMY-AgNPs. The nanoparticles were mainly spherical in shape and 114.76 nm in size; their zeta potential indicated high stability and dispersibility in water. The presence of characteristic functional groups in the FTIR spectra as well as the detection of C, O, and Ag elements in the XPS full-spectrum scan pattern, confirmed the superficial DMY. According to the XRD pattern, DMY-AgNPs had a face-centered cubic lattice structure of silver corresponding to the Miller index of (111) as the main orientation. The DPPH method was used to investigate the ability of DMY-AgNPs to scavenge free radicals, which was found to be comparable to or better than butylated hydroxytoluene or free DMY, with a scavenging rate of 56–92% at concentrations of 0.01–0.1 mg/mL. DMY-AgNPs inhibited *E. coli* and *Salmonella* well, with MICs of 10^−6^ g/L and 10^−4^ g/L, respectively. The antibacterial activity of DMY-AgNPs was shown to be superior and significant to that of DMY and tetracycline, with the inhibition rate increasing with sample concentration. MTT tests were used to assess the anticancer potential of DMY-AgNPs. When compared to DMY, the nanoparticles had a considerably stronger inhibitory impact on HeLa cells and a superior and consistent inhibitory effect on HepG2 cells. Both DMY and DMY-AgNPs inhibited MDA-MB-231 cells quite well, with the latter demonstrating exceptional inhibition (81.44%) at high concentrations.

Silver@quercetin nanoparticles (Ag@QCNPs) were prepared by adding quercetin (QUR) to an ammoniacal silver nitrate solution, resulting in the development of a biocompatible and photostable AIEgen for in situ and real-time monitoring of biomolecules and biological processes [52]. According to the TEM imaging, the nanoparticles were composed of a 35-nm silver core and a QUR shell. The silver core revealed well-organized crystal grains. The fluorescence emission of Ag@QCNPs in different THF/water ratios shifted from 480 nm to 550 nm as the water content increased, with a decrease in fluorescence intensity of less than 10% after irradiation with strong UV light showing resistance to photobleaching. The particle size and fluorescence intensity of Ag@QCNPs were found to be adjustable by varying the amount of QUR used in their preparation, as raising the amount of QUR resulted in an increase in fluorescence intensity and QUR shell thickness. Ag@QCNPs exhibited both aggregation-induced luminescence and the distinct plasma scattering of silver nanoparticles. Ag@QCNPs showed minimal cytotoxicity in HT-29 cells (>95% cell viability). During 60-min co-incubations at 100 μg/mL, the cytoplasm of HeLa cells retained Ag@QCNPs, as confirmed by CLSM images. In the course of in vivo computerized tomography imaging study in mice bearing S180 sarcoma cells, the researchers noted that the Ag@QCNPs accumulated at the highest level (128.6 HU) in the tumor site 2 h after injection before slowly declining over the following 10 h.

**Figure 5:**
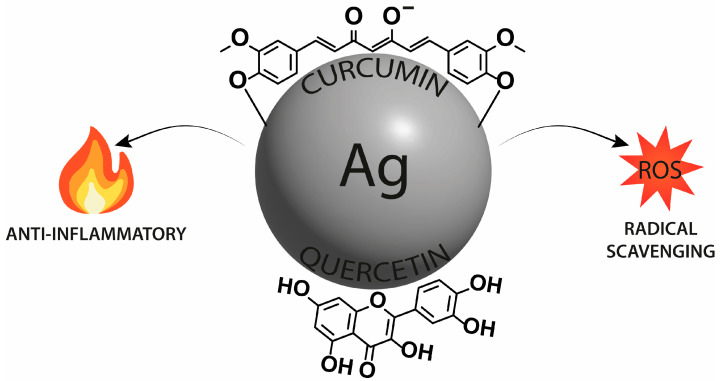
Silver nanoparticles functionalized with curcumin and quercetin and its action.

In the 2022 paper, Kumawat et al. reported on the synthesis of silver nanoparticles double functionalized with curcumin and, among others, quercetin (Cur-Ag^QUR^) to produce a potential anti-inflammatory agent (Figure 5) [53]. The formulation and modification process involved heating and stirring aqueous solutions of curcumin and AgNO_3_, followed by dialysis to remove unreacted molecules and ions. The emerged Cur-Ag was subjected to further surface functionalization with quercetin to produce Cur-Ag^QUR^, which was subsequently dialyzed. The functionalization was achieved through binding interactions mediated by the unique properties of the functionalizing molecules. In quercetin, the reduced form of the polyphenol electrostatically stabilized the metal nanoparticles and attached to the surface of Cur-Ag nanoparticles to form Cur-Ag^QUR^ nanoparticles. The success of the functionalization was indicated by a change in color intensity and the excitation of metal nanoparticle surface plasmon vibrations. The stability and surface charge of these nanoparticles were evaluated using UV-vis spectroscopy, TEM, zeta potential, and DLS measurements. The results showed that Cur-Ag^QUR^ was stable, monodisperse, and had a negative surface charge. The surface functionalization of these nanoparticles resulted in an increase in their hydrodynamic size but did not affect their stability or surface charge. The Cur-Ag^QUR^ nanoparticles were then tested for their potential biological properties, including radical scavenging capacity (RSC), haemocompatibility, and anti-inflammatory effects. The results showed that Cur-Ag^QUR^ had the highest RSC among other tested probes (including bare Cur-Ag, conjugates with isoniazid and tyrosine). The haemocompatibility of the nanoparticles was also found to be good, as the percentage of hemolysis was less than 5% in all tested concentrations. In terms of their anti-inflammatory effects, Cur-Ag^QUR^ inhibited the secretion of pro-inflammatory cytokines from macrophages stimulated by lipopolysaccharide (LPS). The researchers also conducted cell viability studies using the MTT assay on mouse macrophages and found that the nanoparticles demonstrated good cell viability at all evaluated doses, with the Cur-Ag^QUR^ nanoparticles showing the highest viability. In addition, the researchers used the 2′,7′-dichlorofluorescin diacetate assay to assess the potential toxicity of nanoparticles on different organelles via reactive oxygen species (ROS) production and showed that Cur-Ag^QUR^ significantly reduced ROS in macrophages. As far as the anti-inflammatory effects of nanoparticles were concerned, it turned out that they significantly reduced the production of pro-inflammatory cytokines in macrophages. The expression of pro-inflammatory cytokines (such as TNF-α, IL-6, and IL-1β) was raised when treated with LPS. However, when co-treated with nanoparticles, the expression of TNF-α was significantly reduced, which, obviously, could be attributed to the presence of curcumin or quercetin biomolecules on Cur-Ag^QUR^. Silver nanoparticles are known for antibacterial, antifungal, and immunomodulatory activities. Curcumin reveals anti-inflammatory effects due to its structure, which influences transcription factors, cytokines, and protein kinases and inhibits the synthesis of inflammatory molecules such as TNF-α. QUR also inhibits the production of TNF-α and nitric oxide (NO) in murine macrophages. Thus, summing up, double-functionalized nanoparticles can down-regulate pro-inflammatory genes while their biomedical potential can be improved by choosing proper biomolecules and nanoparticles.

In their study from 2021, Wang et al. explored the production and characterization of isoorientin-loaded silver nanoparticles (AgNPs-Iso) as well as their stability and possible therapeutic uses [54]. A green synthesis approach was employed to create AgNPs from corn starch. The addition of SC increased AgNP production, as evidenced by the distinctive surface plasmon resonance peak at 403 nm and the color shift from white to brown. Following that, AgNPs were combined with isoorientin (Iso) and centrifuged to produce AgNPs-Iso precipitate with 76.60% loading efficiency, as confirmed by spectrophotometry. AgNPs had a mean size of 65 nm, while AgNPs-Iso had a size of 117 nm. For the characterization of nanoparticles, TEM was used to examine the morphology, whereas FTIR spectroscopy was used to evaluate the functional groups on the surface of nanoparticles. Stability testing revealed that AgNPs and AgNPs-Iso were stable in pH 5–9 and 0–0.30 M NaCl solutions. At lower pH values and higher concentrations of NaCl, aggregation was observed. In the simulated gastrointestinal tract, AgNPs-Iso was shown to be more stable than AgNPs. Moreover, the more stable nanoparticles displayed no changes in Iso retention even after 1 h of UV irradiation. The hemolysis study revealed that while AgNPs and AgNPs-Iso concentrations were reduced, the number of erythrocytes with complete morphology grew, and the erythrocyte hemolysis ratio decreased. The hemolysis ratio of AgNPs-Iso (29%) was much lower than that of AgNPs (about 60%) when the nanoparticles were studied at a concentration of 60 μg/mL. Observations of erythrocyte morphology using an inverted microscope revealed that the AgNPs-Iso nanoparticles are safer than unloaded ones. According to the MTT experiment, AgNPs-Iso had minimal cytotoxicity in HL-7702 human liver cells, suggesting that AgNPs-Iso might mitigate moderate cytotoxicity produced by AgNPs. AgNPs-Iso also inhibited alpha-glucosidase and pancreatic lipase, implicated in the development of type II diabetes and obesity, respectively. These data imply that AgNPs-Iso might constitute a promising therapeutic agent for these disorders.
**To sum up****Pros**The loading of AgNPs with flavonoids induces the biocompatibility of nanoparti-cles and reduces their toxicity.The connections of silver nanoparticles with particular flavonoids revealed a wide spectrum of potential antioxidant, antibacterial, antifungal, antiparasitic, antivi-ral, and anticancer properties.**Cons**Functionalization of nanoparticles with hesperidin was found to cause stability problems, as the zeta potentials of the nanoparticles were closer to neutral [43]. Some of the nanoparticles reveal strong positive zeta potentials, which may cause toxicity [55].The nanoparticles discussed in the presented studies were of relatively small size, <20 nm, which is a poor indicator for biocompatibility [56].

AgNPs conjugated with flavonoids exerted satisfying antibacterial [43,44,45] and antifungal activity [46]. Moreover, when in co-administration with zinc(II) ions they exhibit antiviral activity [47]; furthermore, when combined with gold, antiparasitic activity was observed [48]. Silver@quercetin nanoparticles were proposed as luminogens for the monitoring of biomolecules [52] due to their ability to accumulate in the tumor site, whereas silver@myricetin [50] might be useful in the treatment of human colorectal cancer. Double-functionalized AgNPs with curcumin and quercetin seem to offer a promising anti-inflammatory nanocure [53]. AP-based silver nanoparticles revealed cytoprotective activity on mouse hepatocytes and increased the expression of liver antioxidant enzymes SOD and GPx [49]. Isoorientin-loaded silver nanoparticles (AgNPs-Iso) appeared to inhibit enzymes implicated in the development of type II diabetes and obesity [54], whereas silver nanoparticles (AgNPs) functionalized by DMY demonstrated antioxidant, antibacterial, and anticancer properties [51]. The summary of the data related to connections of silver nanoparticles with flavonoids, which were presented in this section, is included in Table 1.

### 3.2. Gold Nanoparticles

**Figure 6:**
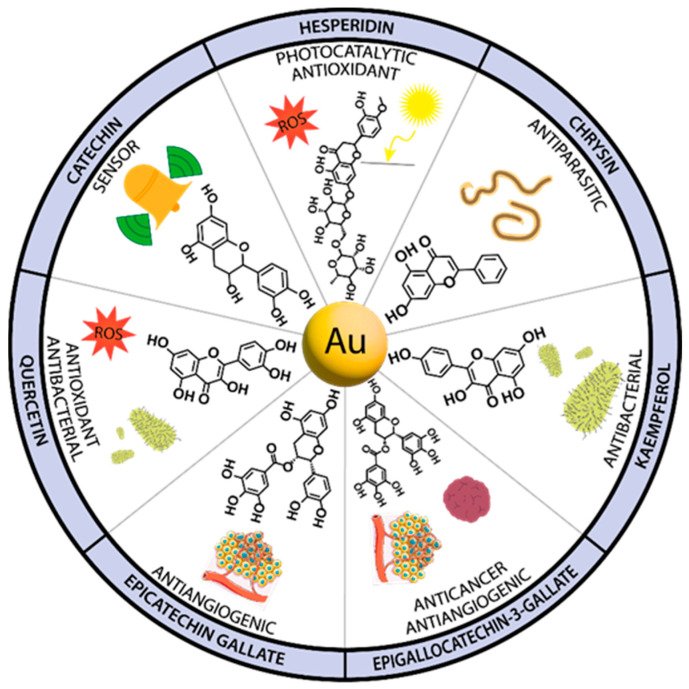
Spectrum of flavonoid-gold nanoparticle conjugates and hybrids with their most striking biological features.

Gold nanoparticles (AuNPs), similarly to AgNPs, have been eagerly studied since their discovery. This is mainly due to the ease of their preparation, simple surface functionalization, and a wide array of potential uses, such as, but not exclusively, in the medical sciences [57]. Additional application of flavonoid compounds improves the biological effects exerted by gold metallic particles, providing potential therapeutics with a range of properties (Figure 6). Of great interest are the potential antibacterial, antioxidant, antiparasitic, antiangiogenic, and anticancer properties of such nanoparticles. In this regard, the nanoparticles were functionalized with chrysin, kaempferol, quercetin, epigallocatechin gallate, procyanidins, 4′,7-dihydroxyflavone, metal-phenolic networks, and green tea polyphenols. In addition, a gold nanocluster was applied for the sensitive and selective detection of dopamine, whereas hesperidin isolated from orange peel was used in the synthesis of gold nanoparticles, which was applied both as an antioxidant and a photocatalyst for the treatment of industrial wastewater.

#### 3.2.1. Gold Nanoparticles in Therapy of Infectious Diseases

In 2021, Alhadrami et al. composed gold nanoparticles (GNPs) coated with chrysin (CHY), kaempferol, and QUR, to boost their antibacterial action against Gram-negative bacteria [58]. GNPs were made by combining an aqueous tetrachloroauric acid (HAuCl_4_) solution with an aqueous GSH solution, adjusting the pH to 8 with NaOH, and then adding NaBH_4_ until a ruby-red hue emerged. After centrifugation, the freshly formed GSH-GNPs were incubated with various flavonoids, allowing them to attach to the carboxylate groups of GSH and overlay the GSH-coated GNPs. The binding effectiveness of flavonoids to GSH-coated GNPs was 80%, 71%, and 41% for quercetin, kaempferol, and CHY, respectively, indicating a correlation between the level of hydroxylation of flavonoids and their binding efficiencies. Flavonoids were effectively coated on the GNPs, which was confirmed by UV-vis spectroscopic evaluations with characteristic changes in their plasmon resonance absorption band, FTIR spectra, as well as powder XRD patterns with characteristic peaks for Au, GSH, and flavonoids. GNPs revealed an average particle size of 4.1–35 nm, with a monodisperse, homogenous spherical, and hexagonal prism-like form. EDX proved the presence of Au in various percentages in each sample, up to 24.71% in GNP-quercetin, and the presence of C and O in adequate proportions to corroborate the loading of flavonoids. In the in vitro evaluations, GNP-quercetin was the most effective against all Gram-negative bacteria tested, with particularly strong action against *E. coli*, *P. aeruginosa*, and *P. vulgaris* (MIC 30 μg/mL) and *K. pneumonia* (MIC 60 μg/mL). GNP-kaempferol showed a strong antibacterial effect against *E. coli* and *P. vulgaris* (MICs of 60 and 30 μg/mL, respectively), but not against *P. aeruginosa* and *K. pneumonia* (MICs of 240 and 120 μg/mL, respectively). GNP-CHY inhibited *E. coli* well (MIC 60 μg/mL) but was less effective against the other microorganisms tested (MIC > 240 μg/mL). GNP-quercetin conjugate was chosen for the study of the antibacterial action. The TEM pictures evidently demonstrated disrupted membranes and the presence of GNP-quercetin nanoparticles inside *E. coli* and *P. aeruginosa* cells. Such an effect was not noted for uncoated GNPs. When flavonoids were docked to the binding site of DNA gyrase, they acquired greater docking scores with the subunit Gyr-B than with Gyr-A. Quercetin was the most potent (IC_50_ 0.89 μM), and CHY was the least potent (IC_50_ 3.91 μM), which indicates that the degree of hydroxylation of the flavonoids appears to be associated with their inhibitory activity on Gyr-B. In silico experiments were performed to understand better the antibacterial activity of flavonoids. The authors built a model of a 5 nm GNP coupled to GSH molecules and docked each flavonoid against it. According to the study, quercetin and kaempferol presented more stable interactions with GSH than CHY. The authors also investigated the effect of each flavonoid on the bacterial outer membrane, discovering a strong correlation between the flavonoids’ growth inhibitory action and the local increase in membrane fluidity. Overall, quercetin was found to be the most effective of the three flavonoids studied.

**Figure 7:**
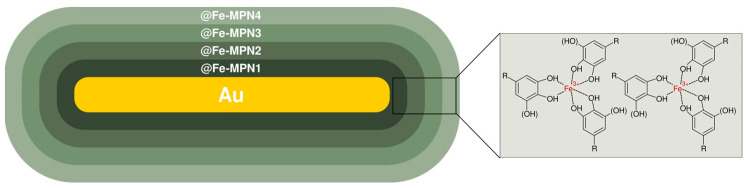
Metal-phenolic networks rendering photothermal bactericidal nanoparticles (GNRs@MPNs) with phenolic fragments such as epigallocatechin gallate, procyanidins, and tannic acid.

In another study, Zhang et al. managed to functionalize gold nanorods (GNRs) with metal-phenolic networks (MPNs), rendering photothermal bactericidal nanoparticles (GNRs@MPNs) (Figure 7) [59]. As phenolic motifs, epigallocatechin gallate (EGCG), procyanidins (OPC), and tannic acid (TA) were chosen. To generate starting nanorods, the authors employed the seed-mediated growth method delineated by Nikoobakht and El-Sayed [60] as well as the conformed procedure of Fu et al. [61]. According to the technique, the solutions of HAuCl_4_ and cetyltrimethylammonium bromide (CTAB) were stirred together and then injected with ice-cold sodium borohydride (NaBH_4_) solution to provide the brownish seed solution that further was subjected to incubation. The growth mixture was prepared on the basis of a CTAB-sodium oleate binary surfactant mixture with AgNO_3_ and consecutive addition of HAuCl_4_. The resulting solution was adjusted with HCl to an appropriate pH level, injected with the ascorbic acid solution, combined with a small amount of the seed solution, and then incubated to undergo centrifugal purification of GNRs later. The obtained supernatant was concentrated and incubated in polyphenolic solutions to replace capped CTAB. To encapsulate MPNs, obtained GNRs were mixed with ultrapure water, sequentially injected with polyphenol solution of a specific kind (EGCG, OPC, or TA) and with iron chloride hexahydrate (FeCl_3_·6H_2_O) solution, then combined with Tris-HCl buffer (pH 8). The forthcoming centrifugation and redispersion with water resulted in monolayered GNRs@MPNs (GNRs@MPN1)–GNRs@Fe-EGCG1, GNRs@Fe-OPC1, and GNRs@Fe-TA1 for each particular polyphenolic substrate. Later, the encapsulation process was repeated on GNRs@MPN1 as a precursor leading to GNRs@MPN2/3/4. The 808 nm laser thermography of new assemblies revealed a good synergy between GNRs and MPNs components in terms of temperature increment compared to GNRs alone, which could be reflected in the following manner: @Fe-OPC > @Fe-TA > @Fe-EGCG > GNRs. The in vitro antibacterial studies were conducted with and without NIR irradiation on *S. aureus* and *E. coli* O157:H7 cultures involving unclad GNRs as well as their three-layered polyphenolic compositions. The 808 nm light solely could not induce any apparent damage to bacterial samples. Treatment with bare GNRs caused negligible suppression to *S. aureus* (about 6.4% according to fluorescence imaging) and moderate detriment to *E. coli* O157:H7 (16.23%). However, the NIR laser application increased the antibacterial rate of GNRs to 30.07% and 69.8%, respectively. The irradiated @Fe-OPC3, @Fe-TA3, and @Fe-EGCG3 expressed 98.6%, 88.6%, and 85.4% respective suppression towards *S. aureus*, along with 99.8%, 90.5%, and 85.07% suppression in the case of *E. coli* O157:H7. What is interesting, the authors studied the antibacterial behavior of goldless MPNs towards *S. aureus* colonies, revealing the suppressing activity of 52.1%, 40.5%, and 33.2% for OPC-MPN, TA-MPN, and EGCG-MPN respectively. Further, a mice model with methicillin-resistant *S. aureus* (MRSA)-infected wounds was utilized, allowing for the investigation of the effectiveness of GNRs@MPNs in a more realistic and relevant setting. Treatment of an artificially inflicted wound with @Fe-OPC3 resulted in a slower healing process, with a 32.6% reduction in wound area after 8 days, mostly due to the chemical sterilization properties. However, the use of GNRs and NIR irradiation led to the formation of a scab and a 46.5% reduction in the wound area, indicating that the GNRs had a moderate ability to convert light into heat, which aided in the healing process. Treatment with NIR-irradiated @Fe-OPC3 significantly accelerated wound healing, resulting in an 88.24% reduction in the wound area after 8 days. This bactericidal outcome likely occurred due to the synergistic photothermal effect of @Fe-OPC3. A plate counting assay also showed a significant reduction in the presence of MRSA bacteria in the wound tissue after treatment with irradiated @Fe-OPC3. The H&E staining histological analysis was used to study the effects of GNRs and @Fe-OPC3 on bacteria and wound healing. GNRs without NIR irradiation revealed weaker performance in wound healing, while NIR-irradiated GNRs partially regenerated epidermis tissue. In contrast, @Fe-OPC3 treated with NIR irradiation was successful in promoting the generation of intact epidermal layers in wound tissue, demonstrating the distinguishable photothermal potential for both antibacterial activity and wound healing as a promising candidate for use in these applications. The biosafety of GNRs@MPNs was extensively tested both in vitro and in vivo. The cytotoxicity was assessed on 3T3 cells, and the nanostructure appeared not to cause a significant decrease in cellular viability at concentrations of up to 100 ppm, indicating its non-toxic nature. According to blood compatibility evaluations with murine red blood cells, GNRs had high hemolysis at 89.79%, indicating poor blood compatibility. @Fe-EGCG3 and @Fe-OPC3 had a low hemolysis ratio of approximately 0% and 1.50%, respectively, while @Fe-TA3 did not present satisfactory blood compatibility at all. Moreover, no significant changes were observed during mice body weight assessment after the injection of GNRs@MPNs (with and without NIR), indicating their favorable biosafety. H&E staining on main organ tissues (including heart, liver, spleen, lung, and kidney) showed that GNRs caused toxic effects, while GNRs@MPNs had no notable abnormalities or damage and were safe for wound healing, even when irradiated. These results demonstrated the effectiveness and biocompatibility of GNRs@MPNs as bactericidal agents.

In 2021, Das et al. developed quercetin-conjugated gold nanoparticles (QuAunps) as a promising agent of antibacterial and antioxidant features [62]. To obtain nanoparticles, a chloroauric acid solution was mixed with a Tween 80 solution, then mixed with a cyclomixer and combined with a methanolic QUR solution. The mixture was then sonicated, and the progress of the reaction was monitored using UV spectrophotometry. The nano-gold was then separated by centrifugation, washed, and stored for further use. The characterization of the nanomaterial was performed using DLS (a hydrodynamic diameter of 62 nm), HRTEM (spherical shapes with a narrow particle size distribution and an average size of 30 nm), energy dispersive X-ray (EDX), and FTIR. The antioxidant activities of QuAunps were investigated using the measurement of the ability to inhibit oxidation of brilliant cresyl blue. QuAunps presented a concentration-dependent activity manifested by 42% and 67% antioxidant activities at 8 μg/mL and 45.8 μg/mL, respectively. Antibacterial assays showed that QuAunps had a lower MIC (7.6 μg/mL) against *E. coli* than free QUR (24.7 μg/mL) and control citrate-capped Aunps (50.8 μg/mL). The authors also examined the cytotoxicity of QuAunps, QUR, and ciprofloxacin (a control drug) in Vero cells and concluded that QuAunps had the highest biocompatibility with a CC_50_ value at 956.8 μM. The MBC of QuAunps was 10.5 g/mL. On agar plates treated with QuAunps, bacterial growth was noted below the MBC after 24 h, but not at or above the MBC. In addition, the dye uptake efficiency of QuAunps was measured at 48.37%, which was significantly higher than those noted for the ciprofloxacin group (19.39%) and the bacterial control group (7.33%). Dye inclusion assays showed that QuAunps damaged the bacterial membrane, resulting in increased dye uptake. The TEM micrographs were applied for the study of QuAunps interaction with the bacterial surface.

The potential of selected functionalized gold nanoparticles has also been studied against selected parasites. In the previous chapter, the antileishmanial activity of 47DHF-functionalized silver nanoparticles (Ag-47DHF) was discussed [48]. In the same paper, the authors also studied the potential of Au-47DHF nanoparticles, which were synthesized by adding 47DHF in varying ratios to HAuCl_4_·3H_2_O solutions while stirring the mixture at a constant temperature and allowing the reaction to continue for 10 min after the color change had occurred. The Au-47DHF nanoparticles were oval and spherical in form, with a zeta potential of 60 mV, and had a size range of 6–7 nm, with an average size of 5.8 nm. UV spectroscopy, the high-resolution transmission electron microscope (HRTEM) pictures, DLS investigations, selected area electron diffraction (SAED) patterns, and FTIR analyses confirmed the structures of both nanoparticles. The nanoparticles had drug loading (DL) efficiencies of 80.79% and 64.13%, respectively. The release of 47DHF from the gold nanoparticles was gradual; at pH 7.4 and 5.8, its cumulative drug release was 14.95% and 57.97%, respectively. Au-47DHF was very effective against both developmental stages of the parasite with IC_50_ values against promastigotes and axenic amastigotes at 0.1226 µg/mL and 0.115 µg/mL, respectively. With IC_50_ values of 0.121 µg/mL, Au-47DHF demonstrated antileishmanial action and targeted intracellular amastigotes. When compared to the infected control, the nanoparticle-administered macrophages generated reduced nitrite concentrations, indicating a decrease in parasite burden. The quantity of ROS (the key trigger of apoptosis induction for 47DHF) formed in Au-47DHF was more than that noted in control H_2_O_2_. In another study related to the antiparasitic action of gold nanoparticles, Raj et al. developed and tested novel chrysin-gold nanoparticles (CHY-AuNPs) and studied their potential against leishmaniasis [63]. CHY-AuNPs were synthesized by mixing CHY and HAuCl_4_⋅3H_2_O solution in a 1:2 ratio. The synthesis of nanoparticles was optimized through parameters such as temperature, pH, and concentration of metal ions, resulting in smaller and more stable nanoparticles as indicated by UV spectra. An acidic pH and high temperature were found to be favorable for the synthesis of CHY-AuNPs. Biosorption, mediated by the hydroxyl group of CHY, occurred at the surface of the nanoparticles due to the interaction between positively charged functional groups and anionic gold ions. The characterization of nanoparticles was performed using UV-vis, and HRTEM micrographs (an average size of 20 nm and a spherical or oval shape), whereas the high crystallinity was confirmed by the clear dotted lattice fringes observed in selected area diffraction SAED patterns. The nanoformulation was found to have a high DL efficiency of approximately 91% for CHY. During the drug release study, it was found that a higher percentage of the drug was released in neutral pH conditions (40–90%) compared to acidic pH (0.9–30%). When tested for oral efficiency, the release of the drug was minimal in highly acidic conditions, but increased significantly at pH 6.8 and 7.4, with approximately 30% and 80% of the drug released, respectively. The in vitro antileishmanial activity of CHY and CHY-AuNPs was assessed by measuring the reduction in *L. donovani* burden in infected macrophages after 48 h of treatment. The results showed that CHY-AuNPs were more effective in the reduction in the parasite viability with an IC_50_ value of 0.8 μg/mL, compared to CHY with an IC_50_ value of 2.19 μg/mL. This suggests that the enhanced efficacy of the drug conjugated with AuNPs may result from targeted delivery.

#### 3.2.2. Gold Nanoparticles in Anticancer Therapy

In a recent work from 2022, Cunha et al. reported the improvement of the EGCG’s efficacy through its conjugation with gold nanoparticles (AuNPs), which led to two nanosystems based on AuNPs [64]. The chitosan (CHI) mediated nanoparticles (EGCG-ChAuNPs) were synthesized by mixing an aqueous solution of chloroauric acid with CHI and heating until a red suspension was obtained and subsequent conjugation with EGCG via carbodiimide-mediated cross-linking (EDC/NHSS). Meanwhile, to prepare the cysteamine-mediated nanoparticles (EGCG-CystAuNPs), the Turkevich method was employed, followed by subsequent functionalization with cysteamine. The resulting CystAuNPs were conjugated with EGCG through EDC/NHSS coupling and stabilized by tween 80. The obtained nanoparticles were characterized using techniques such as UV-vis spectroscopy, DLS, zeta potential study, TEM, and Attenuated Total Reflectance Fourier Transform Infrared spectroscopy (ATR-FTIR). The nanoparticles turned out to exert antioxidant and cytotoxic effects in pancreatic cancer cells, with EGCG-ChAuNPs and EGCG-CystAuNPs inducing 50% cell growth inhibition at EGCG concentrations of 2.2 and 3.7 μM, respectively. The EGCG alone required a concentration of 23 μM to achieve the same level of cytotoxicity. Caspase-3 activity assay also showed that the conjugation of EGCG with AuNPs enhanced apoptosis in the cancer cells compared to EGCG alone. The authors concluded that AuNP complexes could be used as delivery carriers to increase EGCG antioxidant activity in cancer tissues. In the study from 2021, Panda et al. examined the antiangiogenic properties of green tea polyphenols (GTP) combined with gold nanoparticles (GTP-AuNPs) [65]. The nanoparticles were prepared by adding a solution of EGCG or (−)-epicatechin gallate (ECG) to HAuCl_4_ in deionized water and stirring. The obtained red mixture was then centrifuged, washed with DI water, and stored for future use. AFM, UV-vis spectroscopy, and DLS were used to characterize the size, shape, and stability of GTP-AuNPs, which appeared to be influenced by the solubility of the polyphenols in the aqueous medium, with ECG-AuNPs revealing a larger diameter due to the formation of agglomerates and EGCG-AuNPs being more stable due to their higher solubility. Unlike the wild angiogenin (Ang), the protein used in this study had a 6His-tag at the N-terminal, increasing the count of amino acid residues to 144 (from 123) and the molecular weight to 16 kDa (from 14 kDa). Using an agarose gel-based assay, the inhibitory efficacy of ECG and EGCG against the ribonucleolytic activity of Ang was investigated, and it was discovered that EGCG was a significantly more potent inhibitor than ECG, with a relatively higher band intensity. The binding of ECG and EGCG was similar to binding to ribonuclease A, and both flavonoids decreased Ang catalytic activity through interaction with Lys 40 but did not directly alter the ribonucleolytic site. ECG and EGCG bound to Ang noncompetitively, with the gallate moiety (ring D) playing an essential role. The docking results were supported by the in vitro and in vivo effects of ECG and EGCG on Ang, indicating that the polyphenols inhibit Ang via an allosteric manner of inhibition. Kinetic experiments showed that all polyphenols bound to an allosteric site on Ang in a noncompetitive manner, whereas capped AuNPs demonstrated competitive binding with very low inhibition constants (Ki~4 μg/mL). Fluorescence quenching tests and binding constant estimates further confirmed the capacity of GTP-AuNPs to bind to the active site on Ang. The GTP-AuNPs also suppressed the in vivo angiogenic response in the chick chorioallantoic membrane (CAM) experiment. These findings show that ECG- and EGCG-capped AuNPs possess better antiangiogenic properties than their free analogs.

#### 3.2.3. Other Types of Gold Nanoparticles Activity

In 2022, Liu et al. developed a nanoprobe using a CT-functionalized gold nanocluster (C-Au NC) for sensitive and selective detection of dopamine (DA) [66]. To synthesize C-Au NCs, glutathione (GSH)-Au NCs were first prepared by mixing hydrochloride gold and GSH in ultrapure water, heating the mixture, and purifying the resulting GSH-Au NCs through ethanol precipitation. Catechin was then modified with the chemical linker 4-((2,5-dioxopyrrolidin-1-yloxy)carbonyl) phenylboronic acid (BE) to create a BE-CT complex. This complex was mixed with GSH-Au NCs solution and phosphate buffer (PB), and the mixture was stirred at room temperature for 12 h. The resulting C-Au NCs were obtained through ultrafiltration and stored at 4 °C for further testing and analysis. Characterization techniques, including UV-vis absorption spectra, FTIR, XPS, steady-state and time-resolved fluorescence, DLS, and HRTEM, were used to confirm the successful synthesis of the C-Au NCs. The resulting C-Au NCs were monodisperse with a spherical size of 1.7 nm and a hydrodynamic diameter of 3.6 nm. The surface functionalization of CT did not significantly affect the maximum emission wavelength or fluorescence lifetime of the Au NCs but did lead to an increase in the hydrodynamic diameter. The DA detection mechanism was based on the formation of azamonardine (proved by electrospray ionization-mass spectrometry spectra) via the selective chemical reaction between resorcinol fragment of CT and DA, which leads to enhanced fluorescence emission. The chemical linker used to construct the C-Au NC nanoprobe did not affect the CT-DA reaction, as demonstrated by the fluorescence emission spectra of CT-DA and BE-CT-DA solutions. The optimal pH for the reaction between CT and DA to generate azamonardine compound was found to be 9. The surface CT density of C-Au NCs affected the DA sensing performance, with the reaction kinetics increasing with a higher GSH/BE-CT ratio. The reaction rate was slower than previous reports due to the low reactant concentrations used in this work, as well as the hindering effect of the surface GSH ligand. To ensure the completion of the reaction, the chosen conditions for constructing the ratiometric DA sensing platform were a GSH/BE-CT ratio of 15, pH 9, and a reaction time of 1 h. The sensitivity of the nanoprobes was evaluated by recording the fluorescence emission spectra after adding different concentrations of DA. The test showed a good linear relationship with the DA concentration in the range of 0 to 500 nM, with a detection limit of 1.0 nM. The reproducibility of the nanoprobes was also tested, resulting in a low relative standard deviation of 2.5%. The accuracy of the DA detection was verified through HPLC characterization with a UV detector. The selectivity of the nanoprobes was evaluated in the presence of various interferents and found to only enhance the I461/I560 ratio in the presence of DA. The practicality of the platform for DA detection was demonstrated through testing in urine and cell lysate samples. C-Au NC was not able to detect DA in urine samples but had satisfactory recoveries and low relative standard deviation values when tested by a standard addition method. When tested in cell lysate samples, the platform showed good accuracy, as indicated by favorable recoveries and low relative standard deviation values. According to the results, the platform seems adequate for detecting DA in complex biological media.

**Figure 8:**
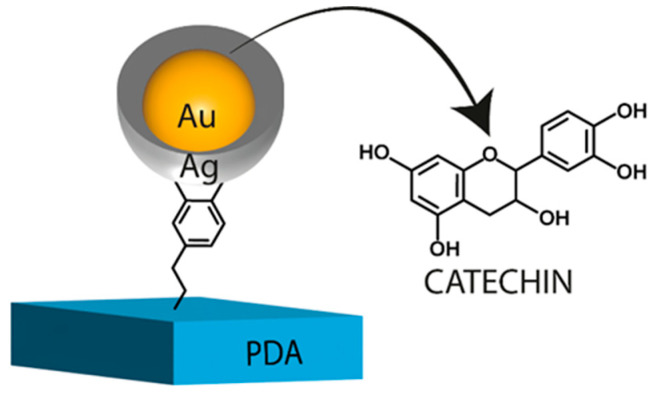
Simplified nanostructured Ag/Au adhesive film.

In another study, Pradhan et al. investigated the potential of hesperidin isolated from orange peels in the synthesis of gold nanoparticles and their use as both an antioxidant and a photocatalyst for the treatment of industrial wastewater [67]. The synthesis of hesperidin gold nanoparticles was achieved by implementing isolated and recrystallized hesperidin into the gold structure using the chemical reduction method with trisodium citrate as a reducing and stabilizing agent while optimizing the conditions. The nanoparticles were characterized by HRTEM, SAED, and FTIR. The photocatalytic activity of hesperidin gold nanoparticles was evaluated on organic dyes and pollutants using sodium borohydride as a reducing agent. The tested chemicals were methyl orange (MO), methylene blue (MB), bromocresol green (BCG), and 4-nitrophenol (4NP). Hes-Au NPs were found to be efficient in degrading these substances in the presence of visible light, with more than 90% degradation observed in all cases. The authors declared that the optimal volume of Hes-Au NPs for degradation of these dyes was 1 mL for MO, MB, and 4NP, and 1.2 mL for BCG, which allowed to degrade of these substances at a level of 88%, 95%, 90%, and 98%, respectively. The optimal concentration of NaBH_4_ was 3 mM for 4NP and 5 mM for the dyes, and the kinetics of the degradation followed first-order kinetics. The dyes degraded the most at 1 mM/0.1 mL, whereas 4NP degraded the most at 2 mM/0.1 mL. In the DPPH and ABTS radical scavenging studies, Hes-Au NPs demonstrated strong antioxidant activity, with IC_50_ values of 37.16 μg/mL for DPPH and 53.57 µg/mL for ABTS. The antioxidant activity of Hes-Au NPs increased with concentration, showing inhibition in the range of 34.8–79% for DPPH and 13.8–74% for ABTS at concentrations ranging from 20 to 100 μg/mL, which is lower than that of referential ascorbic acid with respect to both cases. The nanoparticles outperformed ascorbic acid during the hydroxyl radical scavenging analysis, demonstrating an increase in the scavenging of hydroxyl free radicals from 7.3% to 54% in the range of 20–100 µg/mL (30–52.1% for ascorbic acid). The IC_50_ value in this experiment concluded at 94 µg/mL. Scroccarello et al. developed an adhesive film made of nanostructured Ag/Au and functionalized with CT for reagentless H_2_O_2_ and glucose detection (Figure 8) [68]. The film (PDA@Au-CT@Ag) was created on an enzyme-linked immunosorbent assay (ELISA) polystyrene microplate using a layer-by-layer decoration method that included DA polymerization (to form a PDA layer), the addition of gold nanoparticles (AuNPs), the functionalization of the AuNPs with CT as a reducing agent, and the formation of a silver network by adding AgNO_3_ and NaOH. The resulting film had a reproducible LSPR peak intensity at 405 nm due to the creation of a nano-Ag network and a shoulder at 500–520 nm due to the gold component. The existence of Ag and Au was confirmed by energy-dispersive X-ray (EDX) analysis, which yielded a ratio of 1.15. During the storing assessment, the most promising results were achieved when the films were preserved for one month in MeOH:H_2_O (80:20) and in sodium citrate (10 mM), resulting in 96.5% and 94.2% retention of the original LSPR signal, respectively. In contrast, the PDA@AuNPs film without Ag can be stored at ambient temperature in the dark for one year without significantly changing the LSPR maximum. It was discovered that when PDA@Au-CT@Ag was exposed to H_2_O_2_, its LSPR absorbance changed with the most severe etching occurring at ambient temperature (25 °C), pH 7, within the first 40 min of H_2_O_2_ contact as validated per SEM and EDX. The resultant film was subsequently utilized to quantitatively detect H_2_O_2_ in the range of 1–200 μM, with a detection limit of 0.2 μM. Using GOx as an enzyme, the suggested nanocomposite film was tested for its capacity toward the detection of glucose. The film was discovered to be capable of detecting glucose in the range of 2–250 μM, with a detection limit of 0.4 μM, in a repeatable manner and with a nice linear response. The experiment was performed in a single step and was unaffected by the presence of the enzyme, in contrast to approaches using colloidal dispersions, which frequently require additional stages or enzyme removal procedures to prevent aggregation, precipitation, and enzyme inhibition. The suggested PDA@Au-CT@Ag-based assay was utilized to detect H_2_O_2_ and glucose in several commercial soft drinks, with no substantial signal loss due to any of the evaluated interferents. The assay was shown to be repeatable and accurate, with recoveries ranging from 84–111% for H_2_O_2_ and 83–105% for glucose.
**To sum up****Pros**Gold nanoparticles can be easily prepared and functionalized, and they present significant potential for medical sciences, including antibacterial, antioxidant, an-tiparasitic, antiangiogenic potential, and anticancer properties.The functionalization of gold nanoparticles with flavonoid compounds improves their biological effects. These effects were presented for gold nanoparticles func-tionalized with chrysin, kaempferol, quercetin, epigallocatechin gallate, procya-nidins, 4′,7-dihydroxyflavone, metal-phenolic networks, and green tea polyphe-nols. Due to the photothermal effect, flavonoid-AuNP conjugates reveal significant potential for both antibacterial activity and wound healing.Depending on the conjugation between proper flavonoid and gold nanoparticles, diverse applicability of fabricated systems can be obtained—from antibacterial agents to biosensors. A gold nanocluster was also applied for sensitive and selec-tive detection of dopamine, whereas hesperidin isolated from orange peels was used in the synthesis of gold nanoparticles, which was applied both as an antiox-idant and a photocatalyst for the treatment of industrial wastewater.Despite using structurally diverse flavonoids, no influence on the maximum emis-sion wavelength or fluorescence lifetime of the gold nanoparticles was observed.**Cons**Limited information is known about the long-term toxicity of gold nanoparticles. Some organic functionalizations, such as citrate, might increase toxicity rather than decrease it [69]. Therefore, the toxicity of conjugates should be carefully evaluated.The strong positive potential of some reported herein nanoparticles might raise safety issues, as the feature was correlated with membrane damage [55].In contrast to spherical nanoparticles, gold nanorods are reported to disturb pro-tein structures and reveal reduced mobility - they remain at the injection site, where they might cause damage [69].The competitiveness of the currently developed systems is unclear as there is a lack of studies undertaking this topic.The glucose sensors based on flavonoids [68] still require optimization for better functioning, as commercially used systems are more advantageous [70].

Gold nanoparticles have also been often functionalized with flavonoid compounds to improve their properties and broaden their applications. Gold nanorods with metal-phenolic networks (containing EGCG, procyanidins (OPC), and tannic acid (TA)) [59] used with NIR-irradiation significantly accelerated the infected wound healing and proved their bactericidal effectiveness. Similarly, quercetin-conjugated nano-gold particles exerted antibacterial and antioxidant activity [62] as well as gold nanoparticles, after coating with CHY, kaempferol, and quercetin, boosted their antibacterial action against Gram-negative bacteria [58]. Novel CHY-gold nanoparticles [63] were able to combat leishmaniasis, probably as a result of targeted delivery, whereas GNRs combined with RSV [71] revealed promising but less effective than antileishmanial amphotericin B activity, potentially due to the ROS generation. The CHI- and cysteine-mediated nanoparticles [64] conjugated with EGCG (EGCG-ChAuNPs and EGCG-CystAuNPs) induced cell growth inhibition of pancreatic cancer cells and as delivery carriers increased EGCG antioxidant activity in cancer tissues. Similarly, the delivery of RVS to PANC-1 cancer cells of PDAC was improved by attaching it to gold nanoparticles (GNPs) [72]. Moreover, GTP combined with gold nanoparticles exerted the antiangiogenic properties of (GTP-AuNPs) [65], useful in cancer treatment. C-Au NC can also be used for the detection of DA [66] in complex biological media. Interestingly, gold nanoparticles combined with hesperidin showed antioxidant and photocatalytic potential for the treatment of industrial wastewater [67]. A film composed of nanostructured Ag/Au functionalized with CT can be useful for reagentless H_2_O_2_ and glucose detection. The summary of the data related to connections of gold nanoparticles with flavonoids, which were presented in this section, is included in Table 2.

### 3.3. Metal Oxide Nanoparticles

**Figure 9:**
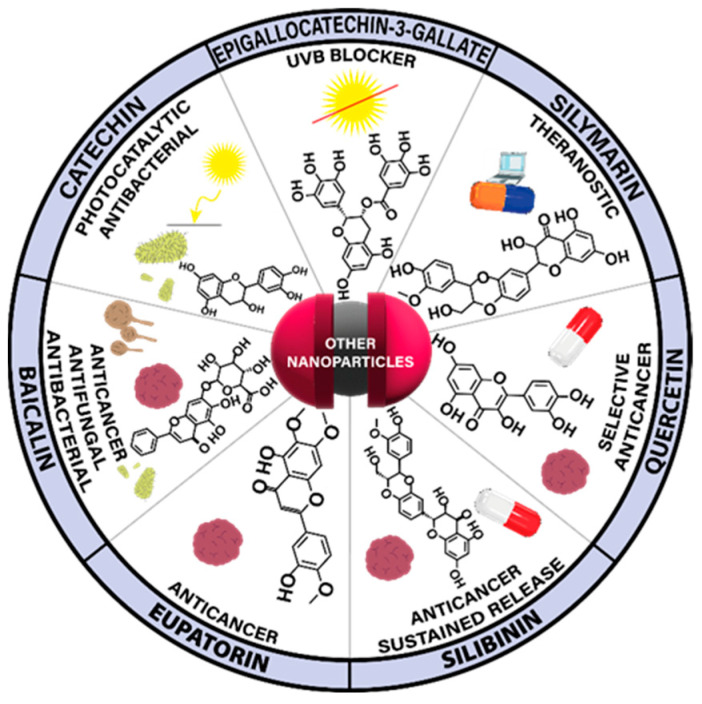
Spectrum of flavonoid-metal oxide nanoparticle conjugates and hybrids with their most striking biological features.

Many studies present connections of flavonoids with metal oxide nanoparticles, mainly zinc oxide, ceria, iron oxide-based, titanium-based, CuO/ZnO, and titanium oxide-gold nanocomposites, which resulted in materials of interesting physicochemical and biological properties (Figure 9). Of great interest are the potential antibacterial, antifungal, antiviral, and anticancer properties of metal oxide nanoparticles as well as their applications as drug delivery platforms for improved osseointegration in orthopedic implants. In this regard, metal oxide nanoparticles were functionalized with quercetin, eupatorin, silibinin, EGCG, baicalin, luteolin, chrysin, morin, icariin hesperetin 7-rutinoside, flavanone-7-*O*-glucoside to study their prospective medical applications.

#### 3.3.1. Iron Oxide-Based Nanoparticles

**Figure 10:**
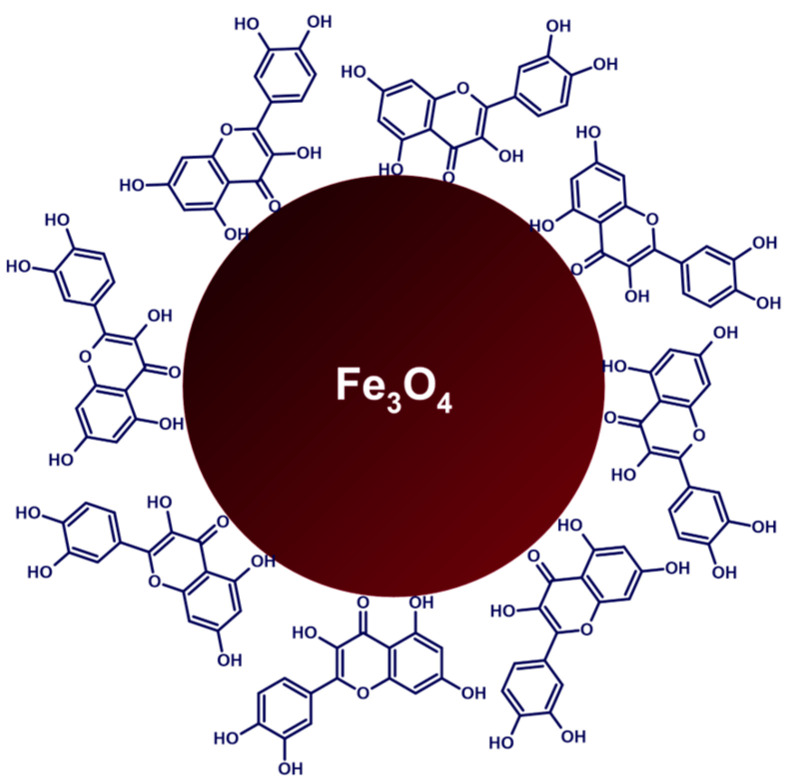
Iron(II,III) oxide nanoparticles clad with quercetin.

In 2022, Askar et al. reported the preparation of quercetin-conjugated magnetite nanoparticles (QMNPs) that exhibited salient antitumor abilities toward breast cancer during the in vitro and in vivo studies [73]. QMNPs derived from iron(II) sulfate solution that was enzymatically converted into iron(II,III) oxide (Fe_3_O_4_) cores of about 11 nm in diameter under treatment by extracellular bio extract from *Aspergillus oryzae* fungus. The obtained MNPs were clad with quercetin, resulting in nanospheres having a diameter of 40 nm (Figure 10). The particles were characterized with the use of UV-vis spectroscopy, FTIR spectroscopy, TEM, and XRD analysis, confirming the positive experimental outcome. In vitro studies were conducted in respect of the ability of QMNPs to inhibit the growth of cancer cell lines MCF-7, HePG-2, and A-459 showing effective inhibition after the 24-h incubation period with IC_50_ values in nanomolar ranges. The authors also described the radiosensitization effect of new particles on the example of MCF-7 cells, demoing the cell decline by 91.2%, compared with the survived cell count after exposure to 6 Gy gamma-ray irradiation (the optimal irradiation dose was estimated experimentally) or 11 nM/mL of QMNPs alone. During the in vivo studies, it was concluded that QMNPs significantly enhanced lateral radiotherapy of the *N*-methyl-*N*-nitrosourea-induced breast cancer in white albino rats through upregulation of pro-apoptotic proteins and downregulation of antiapoptotic proteins of the mitochondrial apoptotic pathway, while preserving nontoxic nature towards hematological, hepatic, and renal markers.

In 2021, Ahmadi et al. developed a nanocomposite of Fe_2_O_3_, CHI, and montmorillonite (MMT) for the encapsulation of QUR as a less harmful alternative to chemical antitumor agents [74]. To obtain the desired Fe_2_O_3_/CS/MMT@QUR, the solution of Fe(NO_3_)_3_·9H_2_O and NH_3_·H_2_O was autoclaved for 10 h to obtain pure Fe_2_O_3_ nanoparticles, which were then mixed with CHI and MMT to be subsequently loaded with QUR. The resulting nanostructure was then crosslinked with glutaraldehyde before being added to a solution of paraffin oil and Span 80. After stabilization with PVA, the nanocarrier was centrifuged from the aqueous solution. According to the XRD study, the Fe_2_O_3_ component exhibited an inverse spinel structure with an average size of 11.79 nm, and the FTIR study indicated the existence of Fe_2_O_3_, QUR, MMT, and CHI in the nanocomposite. As defined by FESEM, Fe_2_O_3_/CS/MMT@QUR had an average size of 148.2 nm, which corresponded to the hydrodynamic radius determined by DLS analysis (161.3 nm). Vibrating sample magnetometry analysis demonstrated the decline in magnetization value after the magnetic nanoparticles were coated with CHI. The NCs exhibited high EE (94%) and pH-sensitive QUR release, with a higher release rate in a slightly acidic environment (pH 5.4). The Weibull model accurately described the drug release data, indicating that the interaction between the carrier and the drug influences the loading and release processes. The NCs also exhibited a regulated release profile, with rapid initial release followed by sustained release. In vitro cytotoxicity tests on MCF-7 cancer cells showed that Fe_2_O_3_/CS/MMT@QUR had lower toxicity compared to free QUR by about 22% due to controllable drug release. As was determined by flow cytometry, the percentages of viable MCF-7 breast cells treated with Fe_2_O_3_/CS/MMT were not substantially different from those in the control group, portraying the carrier as biocompatible and non-toxic. Apoptosis studies for cancer cell lines showed lower viability in the free QUR group, indicating a better function of the QUR-loaded Fe_2_O_3_/CS/MMT nanocomposite, confirming the cytotoxicity experiment, and implying consistency with the release profile. Furthermore, under the presence of Fe_2_O_3_, total apoptotic cells increased by 31.25% as compared to CS/MMT@QUR. In another study, Garfias evaluated the potential of using polyelectrolyte-coated iron oxide nanoparticles as quercetin drug carriers for targeted chemotherapy in ovarian cancer [75]. The synthesis of iron oxide nanoparticles (Fe_2_O_3_) was performed through co-precipitation in an aqueous solution. These nanoparticles were then coated with alternating layers of polyelectrolytes through a layer-by-layer technique. This was performed by adding a volume of an anionic polyelectrolyte solution—poly(styrene sulfonate) (PSS) or sodium carboxymethylcellulose (CMC)—to a suspension of the Fe_2_O_3_ nanoparticles in water, followed by washing and then adding a volume of a cationic polyelectrolyte solution—poly(allylamine hydrochloride) (PAH) or CHI—containing quercetin. This process was repeated to create multiple layers on Fe_2_O_3_ so that quercetin was included in the even layer numbers along with cationic components. The samples were labeled based on the type of polyelectrolyte systems used (synthetic PSS/PAH or natural CMC/CHI, referred to as P or C), the number of layers, and the presence or absence of quercetin in the layers, with QUR indicating the presence of quercetin. The size, structure, and surface properties of the nanoparticles were analyzed using various techniques including DLS, TEM, XRD, FTIR spectroscopy, and vibrating sample magnetometry. The nanoparticles were found to have a mean size of 8.7 nm and a crystallite size of 10.4 nm. Zeta potential measurements and the pH at which the isoelectric point occurred indicated that the polyelectrolytes and quercetin were effectively coated onto the nanoparticles. The nanoparticles showed no cytotoxicity on their own, but when quercetin was included, there was a statistically significant reduction in the viability of human ovarian carcinoma cells. The authors found that the layer-by-layer technique was effective for encapsulating quercetin, with 64.7% efficiency using synthetic polyelectrolytes and 87.7% using natural polyelectrolytes. The authors also observed that the release of quercetin from Fe_2_O_3_ was pH-dependent, with faster release at basic pH and slower release at acidic or neutral pH. Based on the above-mentioned findings, the proposed nanoformulations were proposed for use as targeted drug delivery vehicles for cancer chemotherapy. In 2021, Tousi et al. conducted a study aimed to investigate methoxy poly(ethylene glycol) (mPEG)-b-PLGA coated iron oxide nanoparticles as a carrier of eupatorin in the treatment of prostate cancer [76]. The synthesis started with magnetic nanoparticles (Fe_3_O_4_) fabrication and functionalization with oleic acid. After that, eupatorin-loaded Fe_3_O_4_@mPEG-b-PLGA nanoparticles were generated utilizing a nano-precipitation approach that involved combining Fe_3_O_4_-oleic acid nanoparticles with eupatorin and mPEG-b-PLGA in acetone and stirring the mixture for 10 min. The formulation was then dropwise added to deionized water before being freeze-dried to remove the water. The same method was used to make control samples of Fe_3_O_4_@mPEG-b-PLGA nanoparticles without eupatorin. DLS technique showed that the average particle size was 58.5 nm with a PDI of 0.167. An SAED pattern indicated that the nanoparticles had a consistent spherical form and were thoroughly coated with mPEG-b-PLGA. In the in vitro drug release studies, nanoparticles revealed the rapid initial release of eupatorin (30% over the first 24 h), followed by sustained release over 200 h. The drug content and EE of the nanoparticles were 8.28% and 90.99%, respectively. Eupatorin-loaded Fe_3_O_4_@mPEG-b-PLGA nanoparticles were produced and examined for their capacity to transport eupatorin in a sustained manner to human prostate cancer cell lines DU-145 and LNCaP. The nanoparticles were shown to be efficient in lowering the growth rate of cancer cells in a dose-dependent manner with IC_50_ values of 100 mM and 75 mM for DU-145 and LNCaP, respectively. These values were lower than the IC_50_ values for free eupatorin, showing that the nanoparticles were able to increase the therapeutic benefits of eupatorin. The nanoparticles also caused apoptosis in the cancer cells. The authors also applied flow cytometry to study the effect of free eupatorin and nano-eupatorin on the distribution of cells in different cell cycle stages in DU-145 and LNCaP human prostate cancer cell lines. Free eupatorin produced cell arrest during the G2-M interphase, but nano-eupatorin increased the percentage of cells in the sub-G1 phase, evidencing DNA destruction and apoptosis. The annexin V-PI test revealed that free eupatorin enhanced the number of necrotic and late apoptotic cells in both cell lines. While nano-eupatorin showed similar effects on DU-145 cells, it greatly decreased the number of necrotic cells. There was no significant difference in necrosis or late apoptosis rates in LNCaP cells with either treatment; however, nano-eupatorin was more successful in increasing the proportion of cells undergoing early apoptosis. The annexin V-PI test revealed that nano-eupatorin could considerably lower the rate of necrosis in DU-145 cells while increasing the rate of early apoptosis in LNCaP cells. Nano-eupatorin was considerably more effective, raising the Bax/Bcl-2 ratio in DU-145 and LNCaP to 13.5 and 20.5, respectively. Furthermore, nano-eupatorin enhanced cleaved caspase-3 levels, although no significant change was seen in free eupatorin-treated cells compared to the control group.

In their study from 2021, Takke and Shende used iron oxide nanoparticles to create biocompatible nanopolymeric carriers of PLGA-encapsulated silibinin (SLB-MPNPs) for sustained release in kidney cancer cells [77]. The first synthetic stage involved the addition of H_2_O_2_ to FeCl₂ solution to form a black precipitate, which was separated, washed, and dried to create iron oxide nanoparticles (MNPs). SLB-MPNPs were then created utilizing a two-fold emulsion process that combined PLGA dissolved in acetone, MNPs, and dissolved SLB in ethanol. The resulting primary emulsion was sonicated with aqueous PVA, and the NPs were purified using centrifugation and drying. The PLGA concentration in the SLB-MPNP formulations ranged from 50 to 200 mg, and blank MPNPs were also created. Blank MNPs were measured for particle size and zeta potential and found to have an average particle size of 206.4 nm and zeta potential of −21.1 mV, indicating a stable formulation. SLB-MPNPs M3 (particles composed of 150 mg of PLGA), with a particle size of 285.9 nm and a zeta potential of −14.71 mV, were picked for further investigation. As the PLGA concentration grew, the % EE of the formulations increased from 72.16% to 88.20%. MNPs revealed a greater saturation magnetization (36.35 emu/g) than SLB-MPNPs (12.78 emu/g). The in vitro release analysis of SLB from SLB-MPNPs in PBS at pH 7.4 revealed roughly 48% release after 24 h, with the overall volume of SLB released reaching 65.21% over 2 days. The nanoparticles with the highest encapsulation (200 mg PLGA) demonstrated a delayed and consistent release of 98.04% over 15 days due to drug diffusion from the PLGA core. On A-498 human kidney cancer cells, SLB-MPNPs demonstrated stronger cytotoxicity than plain SLB at all tested doses, with an IC_50_ of 3 μg/mL compared to an IC_50_ of 5 μg/mL for SLB. During the in vivo acute toxicity research, no abnormalities or behavioral changes were detected in the mice, and no tremors, convulsions, salivation, diarrhea, or lethargy were observed. Body mass, food consumption, and water intake did not change significantly between the experimental and control groups, and hematological, biochemical, and organ weight characteristics did not differ significantly either. Histopathological examinations revealed no evidence of tissue injury. For stability testing, the SLB-MPNP formulations were kept at 4 °C preserved from light. The test held for 0, 1, 2, and 3 months revealed no significant alterations, signifying that the formulations remained stable.

In 2021, Qin et al. managed to successfully synthesize photothermally active iron-polyphenol nanoparticles with tunable size and ion content using different polyphenols as a ligand, including EGCG, epicatechin (EC), and proanthocyanidin (PAC) among tested substances [78]. The nanoparticles were synthesized through a sol-gel process that involved dissolving PVP in a mixture of water, ethanol, and ammonia, with the subsequent addition of a specific polyphenol, formaldehyde, FeSO_4_, and eventual autoclaving. Finally, the products underwent dialysis and were isolated through freeze-drying. The DLS investigation revealed that the nanoparticles had hydrodynamic diameters of 21 nm for Fe-EGCG, 27 nm for Fe-EC, and 30 nm for Fe-PAC. All of the nanoparticles were highly dispersed in water and demonstrated good photothermal conversion efficiency varying from 35.2% to 43.6% when irradiated at 808 nm for 10 min. The zeta potentials were noticed to negatively depend on the amount of PVP polymer present. The iron content appeared to impact the photothermal performance of nanoparticles, with higher iron content resulting in better performance, reaching the best results at an iron content of 85.7 mg/g, whereas higher amounts prompted agglomeration of the nanoparticles caused by excess iron acting as a cross-linker. Furthermore, as the size of the nanoparticles decreased, the temperature increased, most likely due to the higher absorption and scattering ratios of smaller particles, resulting in a more efficient light-to-heat conversion. The photothermal performance of colloidal solutions was also affected by both concentration and power density, with higher values leading to increased temperature increases after applying laser while retaining high photostability after multiple cycles of irradiation. Iron-polyphenol colloidal nanoparticles exhibited low cytotoxicity but effectively killed cancer Hela cells under MTT assay through photothermal therapy in vitro when irradiated with lasers. These results were confirmed by in vivo animal studies, which showed a significant increase in the temperature at the tumor site with effective inhibition of the cancer growth in mice after nanoparticles were injected intravenously and exposed to laser NIR irradiation. In terms of biodistribution, the iron content was measured in various organs at different time points after injection of colloidal solution, showing effective accumulation in the tumor due to enhanced permeability and retention effects. The in vivo toxicity of colloidal nanoparticles was assessed using hematological and histochemical analyses, which revealed that the nanoparticles were safe for mice at the current experimental dosage. The hematological analysis found that the white blood cell count was decreased in the colloidal solution group but recovered after 3 days. Histochemical study of multiple organs revealed no evidence of inflammation or injury.

**Figure 11:**
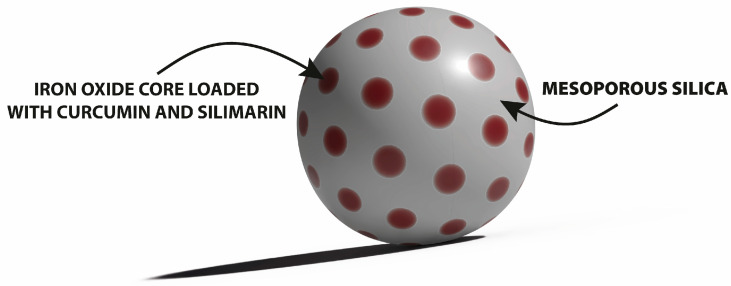
Super-paramagnetic iron oxide nanoparticles coated with mesoporous silica and loaded with the cytotoxic agents curcumin and silymarin.

The study of Sadegha et al. aimed to investigate the potential use of super-paramagnetic iron oxide nanoparticles (SPIONs), coated with mesoporous silica (mSiO_2_) and loaded with curcumin (CUR) and silymarin (SIL), as a theranostic asset for breast cancer treatment (Figure 11) [11]. SPIONs were synthesized via a reverse microemulsion method. Next, the resulting nanoparticles were coated with mSiO_2_ using CTAB and tetraethyl orthosilicate to create mesoporous silica-coated SPIONs (mSiO_2_@ SPIONs), which were then suspended in solutions of CUR and SIL to incorporate the cargo molecules. The average hydrodynamic diameter of SPIONs was 25.50 nm. Following coating with mSiO_2_, the size grew to 57.00 nm but did not alter appreciably after additional processing to remove CTAB. The PDI fell considerably from bare SPIONs to coated and CTAB-free SPIONs. The size and PDI of the SPIONs did not alter much with polyphenols addition. The amount of CTAB employed in the coating process influenced SPION size, with higher levels resulting in bigger sizes. The best quantity of CTAB for adequate size and monodispersity was determined to be 25 mg. As validated by DLS measurements, SEM pictures of the produced NPs revealed that they were spherical and moderately monodispersed, with a size of 60–80 nm. CUR had the highest DL at 0.5 and 1.25 mg/mL, whereas SIL had the highest DL at 1.25 mg/mL. At 0.25 mg/mL, both polyphenols revealed the maximum EE (above 90%). CUR/SIL-loaded mSiO_2_@SPIONs released different amounts of CUR and SIL at different pH values. The MTT experiment on MCF-7 cells revealed that the IC_50_ for the combination of CUR and SIL was lower than that noted for the individual substances. Magnetic resonance imaging (MRI) revealed that the NPs exhibited a high T2-weighted contrast and might be employed in the in vitro diagnosis of early-stage breast cancer.

This kind of potential application of nanoparticles was also presented in another study. Shubhra et al. developed a smart drug delivery system (DDS) marked ICGOx@IO-Dox-EGCG-PPP NPs for multimodal synergistic cancer therapy using magnetic photothermal agents synthesized from iron oxide (IO) nanoparticles with covalently attached indocyanine green (ICG) and glucose oxidase (GOx), coencapsulated with doxorubicin and EGCG inside PLGA nanoparticles, and modified with arginylglycylaspartic acid (RGD) peptides for dual targeting [79]. To make IO NPs, FeCl_2_ and FeCl_3_ were coprecipitated with NH_4_OH at room temperature under vigorous stirring to yield Fe_3_O_4_ NPs, which were subsequently oxidized with NaClO under sonication to produce more stable γ-Fe_2_O_3_ NPs. To stabilize the system even more, citric acid was applied to IO NPs, and massive aggregates were centrifuged from the resultant colloid. Next, to make ICGOx@IO NPs, ICG, and GOx were co-loaded onto the surface of IO NPs. Using a multiple emulsion solvent evaporation process, these NPs were subsequently encased in a PEG–PLGA matrix with Dox and EGCG to form ICGOx@IO-Dox-EGCG-PP NPs. To make ICGOx@IO-Dox-EGCG-PPP NPs, the PLGA NPs in this formulation were tagged with RGD peptide. The hydrodynamic size of the nanoparticles was 209 nm, and their strongly negative zeta potential contributed to their excellent dispersibility and stability. The size distribution was single-point, indicating that the NPs did not aggregate. The nanoparticles were characterized using SEM, TEM (NPs were spherical, with most having a size of 150–180 nm), and FTIR. The saturation magnetization of the ICGOx@IO-Dox-EGCG-PPP NPs was 11.8 emu/g, which was considered sufficient for magnetic targeting. ICGOx@IO-Dox-EGCG-PPP NPs exhibited photothermal properties when irradiated with 808 nm laser light. Irradiation showed an acute increase in temperature to 53.9 °C, while the control samples without ICG showed no significant temperature increase. When external magnets were used for magnetic targeting, the temperature rise reached 55 °C and simultaneously increased the enzymatic activity of GOx. A biphasic release profile was observed for both Dox and EGCG, with an initial rapid release followed by a gradual, sustained release, with a larger rate at pH 5.5 than at pH 7.4, possibly due to enhanced drug solubility at the lower pH and hydrolysis of the PLGA polymer at the acidic pH. At pH 7.4, laser irradiation combined with a magnetic field increased drug release, with up to 73% of Dox and 65% of EGCG released after 48 h. After 5 min of laser irradiation, the PLGA NPs reached the glass transition temperature range of PLGA, promoting drug release as the physical state of PLGA transformed from firm to soft rubbery. Confocal microscopy and flow cytometry were used to investigate the cellular uptake, demonstrating that NPs may successfully transport drugs to B16F10 cells even in the absence of a magnetic field or peptide targeting. When cells were treated with dual-targeted NPs (i.e., with magnet), the highest fluorescence was witnessed, proving that the combination of peptide and magnetic targeting was most successful at delivering the NPs to cells. Single targeting using a peptide or a magnetic field increased cellular uptake over non-targeted ICGOx@IO-Dox-EGCG-PP NPs. Since the magnetic field can only enable NP connection with cells, cell-penetrating peptides directly facilitate NP entrance into cells. The flow cytometry data were supported by a quantitative evaluation of cellular uptake using ICP-MS. DDS was more effective for the reduction in the viability of cancer B16F10 cells than either Dox or EGCG alone, and dual targeting with laser exposure was the most effective for the reduction in the cell viability by 90%. The DDS did not show significant cytotoxicity to non-cancerous HEK293 cells and rendered apoptosis in cancer cells treated with it. ICGOx@IO-Dox-EGCG-PPP NPs were also more effective at declining cell viability in the presence of glucose, possibly due to the enzymatic H_2_O_2_ formation by GOx, and also elevated ROS production. When subjected to both a magnetic field and laser irradiation in vivo, DDS proved efficient at reducing tumor volume and extending mice longevity up to complete tumor eradication, while specimens given free Dox had a lower survival rate, which might be attributed to the negative effects of free Dox. When compared to the control and free Dox groups, the DDS boosted intratumoral H_2_O_2_ concentration and the production of apoptosis-related cytokines in tumor tissues. The NPs accumulative potential in tumor tissues could be expressed in the following order: ICGOx@IO-Dox-EGCG-PPP (+magnet + laser) > ICGOx@IO-Dox-EGCG-PPP (+magnet) > ICGOx@IO-Dox-EGCG-PPP > ICGOx@IO-Dox-EGCG-PP (+magnet) > ICGOx@IO-Dox-EGCG-PP. The researchers discovered that free Dox induced large increases in creatine kinase MB and LDH levels in mouse plasma, signaling cardiac damage, but DDS did not present such an effect. Doxol, a harmful metabolite of Dox, was also found in substantially lower concentrations in the hearts of mice treated with the ICGOx@IO-Dox-EGCG-PPP NPs, which coincided with histological heart tissue damage examinations. Iron concentrations in cardiac tissue were the lowest in mice given the dual-targeted DDS, indicating effective tumor targeting. These findings imply that the introduction of EGCG in the nanoformulations reduced Dox cardiotoxicity by suppressing the activity of carbonyl reductase 1, involved in the synthesis of Doxol. In 2022, Rahmati et al. published a study on the use of QUR-loaded magnetic nano-micelles (QMNMs) as a multifunctional drug delivery platform [80]. QMNMs were prepared using modified oil-in-water emulsion methods. QUR, magnetic nanoparticles (MNPs) of Fe_3_O_4_, and a methoxy poly(ethylene glycol)-block-poly(ε-caprolactone) (mPEG-PCL) copolymer were dissolved in chloroform and added to an aqueous solution containing PVA. The mixture was stirred and homogenized, then allowed to evaporate. The resulting nanoparticles were collected, washed, and dried. The DL and EE of quercetin in the QMNMs were 17.1% and 95.9%, respectively. The stability of the micelles was confirmed by the low critical micelle concentration of 50 mg/L. The characterization of nanoparticles was performed using UV-vis spectrophotometry, DLS measurements (a monodisperse distribution with a polydispersity index of 0.269 and an average hydrodynamic size of 85 nm), and TEM images (semi-spherical shape and an average size of 10–15 nm). The physical stability of QMNMs was assessed by monitoring their particle size over a 90-day period using DLS analysis, and it was found that there were no significant changes in the average size. The successful incorporation of magnetic nanoparticles into QMNMs was confirmed through energy-dispersive X-ray spectroscopy analysis and XRD patterns. The magnetic properties of both MNPs and QMNMs were compared, with the latter showing a reduced saturation magnetization of 12.2 emu/g due to the coating of the nanoparticles with an mPEG-PCL layer. However, both materials displayed superparamagnetic behavior with no remanent magnetization or coercivity at relatively low fields. The observations of drug release from QMNMs represented their sensitivity to pH changes. In general, a burst QUR release occurred in the first 12 h, followed by a sustained release for the next 5 days. At pH 5.3, 28% of the loaded drug was released after 12 h and 55% after 140 h. At pH 7.4, 15% of the drug was released after 12 h and 28% after 140 h. The levels of reduced GSH in isolated rat mitochondria were not reduced in any of the tested groups, signing that oxidation of thiol groups in mitochondrial permeability transition pores did not occur. This suggests that pure QUR, QMNMs, and MNPs did not cause mitochondrial dysfunction in rats. These results were confirmed by lipid peroxydation (LPO) assay.

**Figure 12:**
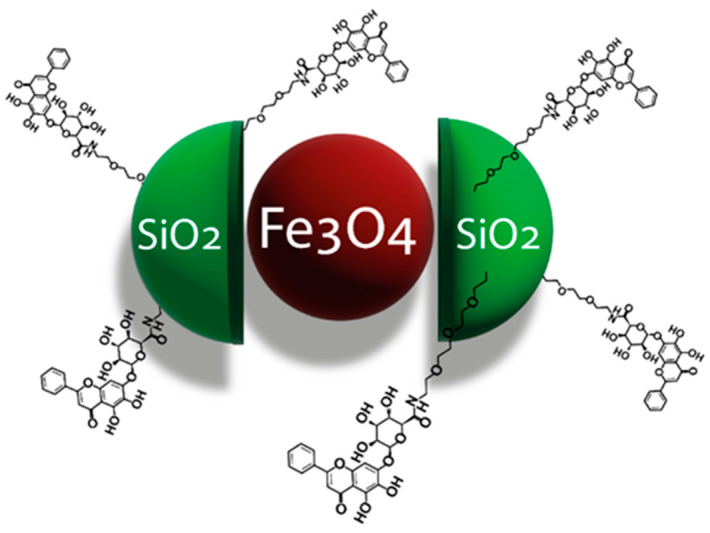
Fe_3_O_4_@SiO_2_ core-shell magnetic nanoparticles with baicalin.

In the study from 2022, Hou et al. synthesized a clickable azido derivative of baicalin (BCL-N_3_) for the functionalization of alkyne-modified Fe_3_O_4_@SiO_2_ core-shell magnetic nanoparticles (MNPs) to create baicalin affinity nanoparticles (BCL-N_3_@MNPs) able to identify proteins that interact with baicalin (Figure 12) [81]. Baicalin was modified by attaching an azide group to its carboxyl via a PEG chain. The resulting BCL-N_3_ was then conjugated with MNPs via an azido-alkynyl click reaction. The study examined whether BCL-N_3_ preserves the biological activity of BCL in human liver microsomes (HLMs) by comparing its inhibitory activity on human carboxylesterase 1 (hCE1). Although both BCL and BCL-N_3_ had similar IC_50_ values, BCL-N3 showed a more potent inhibitory effect at higher concentrations. However, when baicalin was functionalized onto MNPs, its inhibitory activity on hCE1 was even higher at the same concentration as BCL and BCL-N_3_. Researchers optimized the capture efficiency of BCL-N_3_@MNPs on target proteins of baicalin by comparing the number of proteins seized from protein extracts of human embryonic kidney (HEK293) cells. The results showed that 100 nm BCL-N_3_@MNPs had higher capture efficiency and that non-specific protein absorption on BCL-N_3_@MNPs could be reduced by washing the probes. Therefore, the two-hour incubation and washes were defined as optimal conditions that led to the capture efficiency of 19.69 µg/mg for BCL-N_3_@MNPs (versus 4.71 µg/mg for MNPs). A total of 14 proteins were identified from extracts of HEK293 cells through mass spectrometry as interacting with baicalin, including enzymes, transcription regulators, a transporter, a kinase, a translation regulator, and other proteins. The interactions may be related to baicalin’s various pharmacological activities, such as anti-inflammatory and anti-infection effects, and its potential as an anticancer agent. Additionally, baicalin was found to interact with actin-related proteins, which may play a role in its ability to inhibit cancer cell motility, and with peroxiredoxin IV, which may contribute to its antioxidant activity.
**To sum up****Pros**Connections of flavonoids with iron oxide-based nanoparticles resulted in materi-als of interesting physicochemical and anticancer potential, as well as prospective applications as drug delivery platforms.Iron oxide-based nanoparticles were functionalized with quercetin, eupatorin, silibinin, EGCG, and baicalin to study their prospective medical applications.Several types of polymers, e.g., PLGA, PEG, PCL, CMC, and PVP, as well as meso-porous silica, were used for surface functionalization of iron oxide-based nano-particles, which increased the stability of flavonoid-nanoparticle conjugates and impacted the controlled release of organic molecules.The surface functionalization of iron oxide nanoparticles with the use of quercetin reduced their toxicity to healthy cells.Iron oxide-based nanoparticles, especially super-paramagnetic iron oxide nano-particles (SPIONs) loaded with curcumin and silymarin, demonstrated potential as theranostic agents for anticancer treatment.**Cons**For most of the nanoparticles directed against cancers, the MIC/ID50 values were not specified.There are no comparative studies of standard cancer therapy with and without the particular DDS.There is a lack of information about the selectivity index of the synthesized NPs, which is an important parameter allowing us to assess their effectiveness.

Iron oxide nanoparticles have been produced and formulated with flavonoids for applications in the treatment of ovarian cancer [75] after coating with mesoporous silica [11] or in the form of nanocomposite of Fe_2_O_3_, CHI, and montmorillonite (MMT) in breast cancer [74]. Methoxy poly(ethylene glycol) (mPEG)-b-PLGA-coated iron oxide nanoparticles seem to be suitable carriers of flavonoid compounds in the treatment of prostate cancer [76], whereas iron oxide NPs can serve as carriers of PLGA-encapsulated silibinin for sustained release in kidney cancer cells [77]. Iron oxide nanoparticles are also components of drug delivery systems applied in multimodal synergistic cancer therapy [79] and formulations used for the identification of selected proteins [81]. The summary of the data related to connections of iron-based nanoparticles with flavonoids, which were presented in the section, was included in Table 3.

#### 3.3.2. Zinc(II) Oxide-Based Nanoparticles

In a study conducted in 2021, Kollur et al. evaluated the potential anticancer activity of luteolin-functionalized zinc oxide nanoparticles (L-ZnONPs) [82]. L-ZnONPs were made by mixing an aqueous solution of Zn(OAc)_2_ and luteolin, sifting the resulting white precipitate, washing it with ethanol to remove impurities, and calcining the result. Luteolin’s 2′- and 3′-hydroxyls were employed to clad the ZnO nanostructures through the oxidation process with the zinc ions. The obtained nanostructures were characterized using XRD, SEM (nanospheres between 12 and 25 nm in size), and TEM (hexagonal structure with a dimension of roughly 17 nm). L-ZnONPs considerably outperformed individual treatments of luteolin and ZnO in cytotoxicity experiments on MCF-7 cells under hypoxic conditions, lowering the number of viable cells to 15% at a concentration of 40 μM (the IC_50_ value of free L was previously reported as 43 μM for MCF-7 cells). Based on the in silico protein validation, all the proteins chosen for this study (1Q4O, 2FK9, 2LAV, 3PP0, 4RIW, 5YZ0) expressed high levels of favored and allowed residues, allowing them for further molecular interactions. Based on increasing binding affinity, six docked poses for L-ZnONP against a specific protein were achieved, and the results were visualized to investigate interactions between the ligand and protein. The further computations allowed the prediction that L-ZnONPs interact with 1Q4O, 3PP0, and 2LAV proteins via hydrogen bonds with binding affinities of −9.7, −8.3, and −10.1, respectively. The other proteins created less hydrogen bonding with L-ZnONPs. The best docked L-ZnONP poses with the selected proteins featured conserved salt bridges and numerous bonded and non-bonded interactions. L-ZnONPs were assumed to limit MCF-7 cell proliferation via molecular interactions with the human polo-like kinase 1 protein. In another study, Ramalingam et al. investigated ZnO nanoparticles functionalized with quercetin (ZnO@Quercetin) with respect to their anticancer efficacy towards human ovarian cancer cells [83]. ZnO@Quercetin nanoparticles were created by dissolving quercetin in a zinc nitrate solution, refluxing the combination, and then adding KOH. The same approach was used to create a control group of ZnO but without the inclusion of quercetin. QUR was also physically combined with ZnO to generate a different sample in which the two were linked by static contacts, van der Waals forces, or Lewis acid-base interactions. According to SEM and EDS analyses, the produced nanoparticles were evenly dispersed, agglomerated, and devoid of contaminants. Elemental mapping using scanning augur microscopy (SAM) revealed a homogeneous distribution of carbon, zinc, and oxygen in the functionalized ZnO@Quercetin, showing that functionalization was effective. The ZnO nanoparticles produced in this work were discovered to be monocrystalline hexagons with a size range of 12–18 nm and a lattice spacing of 0.21 nm, hinting on the wurtzite type XRD examination revealed the typical peaks of ZnO nanoparticles, while Raman spectroscopy validated the nanoparticles’ structural purity. The surface functionalization of QUR with ZnO nanoparticles did not alter the diffraction peaks or lattice planes substantially, showing that the surface functionalization was effective. Functionalization improved the electrochemical performance and surface area of ZnO nanoparticles, as demonstrated by cyclic voltammetry and Brunauer–Emmett–Teller analyses. The functionalized nanoparticles outperformed the non-functionalized ones in terms of redox behavior, conductivity, and surface area. ZnO@Quercetin showed increased concentration-dependent cytotoxicity against human ovarian cancer cells, with a lower concentration of ZnO@Quercetin resulting in increased cytotoxicity (an IC_50_ was about 10 μg/mL), compared to ZnO or QUR alone. This toxicity was expressed in significant morphological alterations induced in cancer cells, including reduced density, detaching, clumping, and floating. The functionalization of quercetin on the surface of ZnO nanoparticles proved to be more effective at causing cancer cells to lose their structural integrity. ZnO nanoparticles were discovered to promote the formation of ROS in cancer cell mitochondria, as seen by weak fluorescence emission in a DHE staining experiment and verified by spectrofluorimetry analysis. QUR boosted ROS creation as well, albeit to a lower amount than ZnO@Quercetin, which had significant fluorescence emission and the highest ROS generation as compared to control cells. The ZnO@Quercetin also demonstrated substantial anticancer efficacy through the permeabilization and ROS production in the mitochondrial membrane, regulating key proteins involved in the intrinsic apoptotic cascade, thus predisposing apoptosis in human ovarian cancer cells. When QUR and ZnO nanoparticles were combined, the number of apoptotic cells increased much more than when each component was used alone. The dual staining experiment discovered that both ZnO and QUR therapy produced early death in the cells yet showed poorer performance in comparison to the ZnO@Quercetin formulation. Mahalanobish et al. described in their study from 2022 the development of a zinc oxide nanoparticle-based drug delivery medium for the targeted delivery of a natural bioactive compound - CHY, to lung cancer cells [84]. To achieve final ZnO-PBA-CHY formulations, zinc oxide nanoparticles (ZnO NPs) and amine-conjugated ZnO NPs (NH_2_-ZnO) were synthesized first through a series of chemical reactions and mixing steps. BE was activated and then combined with the NH_2_-ZNPs to create the ZnO-PBA nanocarrier. Chrysin was loaded onto ZnO-PBA by adding it to the nanocarrier and stirring the mixture. The resulting drug loading content and EE were measured at 30.56% and 44%, respectively. The stability of the nanoconjugate remained relatively constant in a solution with 10% FBS. The results of UV-vis spectra in a dialysis bag experiment showed that the nanoconjugates partially dissolved, releasing almost 59% of the CHY after 48 h at pH 5.0, while only 14% and 9% were released at pH 6.0 and 7.4, respectively. The synthesized nanohybrids were shown to emit blue 4′,6-diamidino-2-phenylindole (DAPI) and green (FITC) fluorescence when viewed under a fluorescent microscope, implying their potential use as a bio-imaging agent. The intake of ZnO and ZnO-PBA nanoparticles in A549 cells was studied using fluorescence-activated cell sorting analysis, which showed that the ZnO-PBA nanoparticles had greater intake efficacy in the cells compared to the ZnO nanoparticles. This increased intake is thought to be due to the interaction of the PBA-tagged nanoparticles with overexpressed sialic acid receptors on the surface of the cancer cells. The cytotoxicity of ZnO-PBA-CHY was examined on A549 and L132 cell lines using the MTT assay, showing dose-dependent cytotoxicity in the A549 cells at doses ranging from 16.3 to 130.8 μg/mL (an equivalent to 5–40 μg/mL of free CHY). The nanohybrids had a greater cytotoxic impact on A549 cells compared to free CHY or ZnO-PBA and did not show significant cytotoxicity in the normal alveolar epithelial L132 cells. The apoptotic effect of the nanohybrids on A549 cells increased the value of apoptotic cells from 6.54% in the control to 12.44%, 22.52%, and 55.62% after treatment with free CHY, ZnO-PBA, and ZnO-PBA-CHY, respectively. Additionally, the nanohybrid successfully stopped the cell cycle in the G0/G1 phase. These findings imply that the nanohybrid triggers the innate cell death mechanisms, causing cellular apoptosis and cell cycle arrest. Finally, by reducing MMP-2 expression and VE-cadherin expression, the nanoparticles inhibited the migration and invasion of A549 cells.

In another study, a quercetin-functionalized CuO/ZnO nanocomposite (CuO/ZnO@Q) was studied in terms of photocatalytic and biocidal activity [85]. CuO/ZnO@Q was formulated by mixing copper acetate and QUR solution, then adding QUR and zinc acetate. The resultant mixture was washed and centrifuged before being dried (yielding CuO/ZnO@Q) and calcined to provide pristine CuO/ZnO for comparative studies. The pure CuO/ZnO XRD pattern revealed the existence of both ZnO and CuO phases, with peaks matching to polycrystalline hexagonal wurtzite structure of ZnO and the monoclinic secondary phase of CuO. The CuO/ZnO@Q pattern was X-ray amorphous, most likely due to the bounded QUR. The FTIR spectrum of CuO/ZnO@Q nanocomposite indicated the majority of the quercetin peaks but with a modest drop in intensity and change in position, indicating surface functionalization. After 600 °C sintering, the FTIR spectrum revealed no peaks that might be ascribed to QUR, but rather the existence of metal–oxygen bonds typical of CuO and ZnO. The nanocomposite was characterized using TEM, EDX, and UV-vis spectroscopy. The photoluminescence (PL) spectra of CuO/ZnO revealed an emission peak at 434 nm, which was ascribed to excitonic band-to-band radiative emission. PL intensity of CuO/ZnO@Q declined, suggesting efficient suppression of charge carrier recombination and higher separation of electron and hole pairs. When exposed to UV light, the excited electrons from RhB and QUR molecules were transported to the CuO/ZnO conduction band, allowing for more effective charge carrier separation and oxidation of the dye molecules. After 75 min of UV irradiation, CuO/ZnO@Q demonstrated nearly full degradation of RhB (99.91%), whereas pure CuO/ZnO reached only 70.78%. CuO/ZnO@Q caused 99.98% degradation after 90 min, whereas pure CuO/ZnO decomposed the dye to 96.5% over the same period. By improving the capacity of CuO/ZnO@Q to absorb UV light at 256 nm and 365 nm, QUR can boost its photocatalytic activity. The UV absorption spectra showed that the degradation of RhB was enhanced in the presence of QUR. The rate of dye degradation rises when the catalyst concentration (CuO/ZnO@Q or CuO/ZnO) increases from 20 mg/L to 30 mg/L (the optimal catalyst concentration). When the concentration was raised to 40 mg/L, however, the solution became turbid, and the rate of degradation decreased. The rate of dye degradation grows as the dye concentration increases from 20 mg/L to 30 mg/L (the optimal dye concentration). However, the degradation rate decreases when the dye concentration approaches 40 mg/L. The recyclability of the CuO/ZnO@Q was demonstrated through five cycles of 75 min long degradation reaction, decomposing RhB with 99.9%, 97.4%, 95.7%, 95.12%, and 94.6% efficacy in the first to fifth cycles, respectively. This demonstrates just a little decrease in dye degradation % throughout the five cycles, proving the stability and recyclability of the produced NCs. During the biocidal evaluations, CuO/ZnO@Q inhibited bacterial strains (*Escherichia coli*, *Staphylococcus aureus*, *Shigella,* and *Bacillus subtilis*) better than CuO/ZnO. The functionalized nanoformulations also revealed substantial antifungal activity against *Aspergillus niger* and *Candida albicans*, unlike CuO/ZnO. The presence of QUR biomolecules hindered the development of the tested bacteria considerably well.

**Figure 13:**
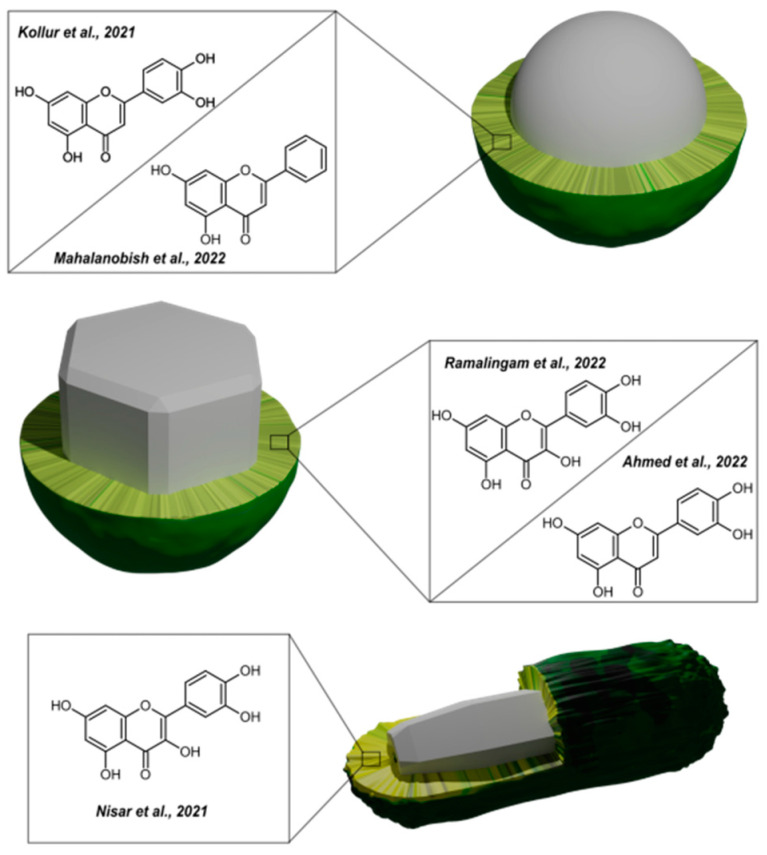
Representative studies on flavonoid-loaded zinc oxide nanoparticles; Mahalanobish et al. 2022 [84], Kollur et al. 2021 [82], Ramalingam et al. 2022 [83], Ahmed et al. 2022 [87], Nisar et al. 2021 [86].

In 2021, Nisar et al. released a study on quercetin-loaded zinc oxide nanoparticles (quercetin@ZnO NPs) as a promising candidate for use in antiphotoaging therapeutics (Figure 13) [86]. To create quercetin@ZnO NPs, quercetin was combined and homogenized with acetone before being diluted in various ratios with as-prepared ZnO NPs. To remove superfluous water, the mixture was ultrasonicated and centrifuged, yielding pure quercetin@ZnO NPs. SEM imaging revealed that quercetin was successfully loaded onto ZnO NPs in the form of flower clusters. The best quercetin/ZnO ratio was determined to be 1:10, and when quercetin concentration grew in different quercetin/ZnO NP ratios, the ZnO structure changed and became less prominent. The optimal quercetin/ZnO mass ratio was discovered to be 10:1, providing the adsorption rate of 90.61% and the loading capacity of 29.35%, potentially allowing for maximum drug penetration. It was noticed that UVA radiation boosted drug release, with 88.71% of quercetin released after 8 h of exposure to a 150 kJ/m^2^ UVA dose. Only 10.29% of quercetin was released from NPs held in the dark over the same time, revealing the stimulatory effect of UVA irradiation on drug release from NPs, most likely due to hydrophobic/hydrophilic transitions. The ability of quercetin to bind iron was demonstrated using cyclic voltammogram studies, and this was confirmed by spectrophotometry through testing different Fe(NO_3_)_3_ concentrations. These findings imply that quercetin can lower ROS generation while also protecting against UV damage. During the test on UVA-exposed HaCaT cells, quercetin@ZnO NPs influenced the reduction of ROS levels and inflammatory factors. These findings suggested that quercetin@ZnO NPs could be employed to minimize the harmful effects of UVA on the skin, such as photoaging. In a cytotoxicity assay, HaCaT cells were exposed to an optimal value of 150 kJ/m^2^ UVA, resulting in approximately 80% cell viability after 24 h. When the cells were treated with different concentrations of quercetin@ZnO NPs at 8 and 24 h after UVA exposure, no cytotoxicity was observed, and the cells showed proliferative behavior, indicating excellent biocompatibility.

**Figure 14:**
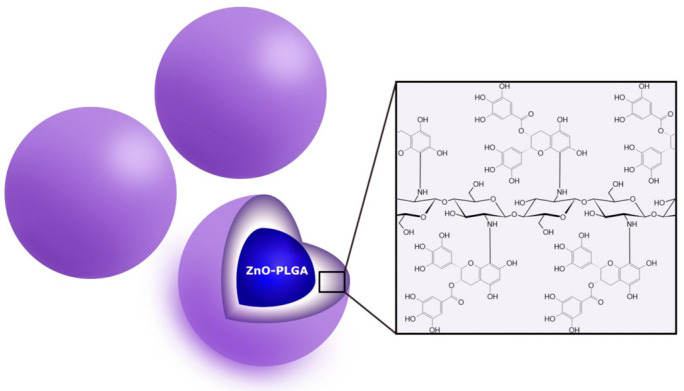
EGCG-CHI/zinc oxide (ZnO)-poly(lactic-co-glycolic acid) (PLGA) nanosystems.

In a related study, Salaheldin et al. investigated the potential use of three innovative (−)-epigallocatechin-3-gallate (EGCG) nanoformulations as natural chemopreventive agents against ultraviolet beam (UVB) radiation-induced DNA damage in keratinocytes [88]. In this study, one of the examined nanosystems was EGCG-CHI/zinc oxide (ZnO)-poly(lactic-co-glycolic acid) (PLGA), which was referred to as Nano 1 (Figure 14). To assemble it, firstly, ZnO nanoparticles were produced via co-precipitation, in which zinc nitrate hexahydrates and sodium hydroxide were combined under stirring. The resulting white precipitate of zinc hydroxide nanoparticles was washed and heated to create a white powder of ZnO nanoparticles. Then, PLGA was dissolved in water with ZnO nanoparticles added to form a ZnO-PLGA suspension. EGCG-CHI was made by mixing ascorbic acid, CHI, and EGCG, to which the ZnO-PLGA suspension was added. The solution was then sonicated and allowed to stir. The resulting EGCG-CHI/ZnO-PLGA was purified through dialysis. Dynamic Light Scattering (DLS) and Electrophoretic Light Scattering (ELS) techniques performed on Nano 1 revealed an average size distribution of 152 nm and Zeta potential of +10 mV. Its EGCG encapsulation efficiency (EE) was 99%, with a loading ratio of 8.4%. Nanoformulation 1 demonstrated slow EGCG release in phosphate-buffered saline (PBS), with a release rate of around 7% even after 24 h. However, in fetal bovine serum (FBS), the release of EGCG from Nano 1 was faster, with a maximum release rate of around 65% in the first hour, followed by a decrease to approximately 40% for the rest of the 24-h period. The stability of nanoformulations was confirmed in this study through the lack of significant change in EGCG concentration during the study period, as well as the stable physical properties, including homogeneity, precipitation, aggregation, and color change. The absorption of UVB radiation by the skin is known to result in the formation of cyclobutane pyrimidine dimers (CPDs) and 6-4 photoproducts (6-4PPs), as well as an increase in the levels of various cytokines and chemokines. To study these effects, the researchers conducted tests on human-immortalized HaCaT keratinocytes. Pretreatment with EGCG and its nanoformulation 1 did not effectively prevent the formation of UVB-induced CPDs or 6-4PPs and did not exhibit significant protective abilities during chemokine/cytokine quantification in the course of in vitro studies. During the in vivo studies, the preventive abilities of nanoformulations were tested on UVB-exposed SKH-1 hairless mice. In terms of protection against UVB-induced DNA damage, nanoformulations showed a 20–45% reduction of UVB-generated CPDs and a 20–48% reduction of 6-4PPs. During the topical application of EGCG and its nanoformulations, the concentration of EGCG in the epidermis was higher for the free EGCG treatment compared to Nano 1. However, the concentration of EGCG in the dermis and hypodermis was higher for Nano 1 treatment compared to the free EGCG treatment. This suggests that the EGCG-CHI/ZnO-PLGA system has higher skin permeability and stability compared to the free EGCG, which may contribute to their protective effect against UVB-induced skin damage.

Another application of zinc oxide nanoparticles was proposed by Ahmed et al., who investigated the potential of luteolin/zinc oxide nanoparticles (Lut/ZnO NPs) to improve insulin resistance and treat non-alcoholic fatty liver disease (NAFLD) in a diabetic rat model [87]. To prepare Lut/ZnO NPs, luteolin was dissolved in water and then combined with zinc acetate and sodium hydroxide under heat and stirring. The solution was washed and exposed to a dose of 10 KGy before being sealed. The properties of the resulting Lut/ZnO nanodispersions were characterized, unveiling a hexagonal shape with a mean size of approximately 172.6 nm as per DLS results (17 nm according to TEM) composed of single crystalline phase of hexagonal ZnO with a mean particle size of 174.7 nm confirmed by the XRD pattern. UV-vis spectroscopy also showed the presence of luteolin with absorbance maxima at 340 and 390 nm. The LD50 test on female rats determined that the safe dose for intraperitoneal injection of Lut/ZnO NPs was 12 mg/kg body weight. The nanodispersions were categorized as safe and effective at reducing blood glucose, insulin levels, and insulin resistance in rats with high-fat diet (HFD)-induced obesity and type 2 diabetes mellitus. The Luteolin/ZnO nano-dispersions also increased the expression of proteins involved in the insulin signaling pathway in the liver, including IRS, PI3K, and AKT, as well as reduced the expression of FoxO1 and its downstream target G6Pase. They were also effective at reducing the expression of SREBP1c, a protein involved in the regulation of lipid and cholesterol metabolism. Lut/ZnO NPs were shown to improve hyperlipidemia in NAFLD and type 2 diabetes mellitus (T2DM) through reducing levels of total cholesterol and triglycerides in both serum and liver tissues, as well as decreasing levels of free fatty acids and increasing levels of high-density lipoprotein cholesterol. It was considered that the antihyperlipidemic effect of Lut/ZnO NPs may indirectly improve insulin resistance by reducing the accumulation of fatty acids and triglycerides in the liver. The nanoparticles reduced lipid peroxidation and improved the antioxidant status in the livers of rats fed HFD and those with T2DM. These effects were indicated by decreases in the levels of MDA and oxidized glutathione as well as increases in the levels of reduced GSH and the expression of heme oxygenase-1. These findings imply that Lut/ZnO NPs may have a protective effect against oxidative stress in the livers of these rats. They also significantly improved liver function in rats with HFD and T2DM, as indicated by a reduction in the activities of liver enzymes—alanine transaminase and aspartate transaminases, manifesting a possible protective effect on the liver. As per a histopathological examination, treatment significantly improved liver damage in rats with HFD and T2DM, as indicated by a reduction in the presence of fatty deposits, necrosis, and hyperplasia of Kupffer cells in the liver tissue, along with an improvement in the regular arrangement of hepatic cords and a decrease in intracellular micro-vesicular steatosis and dilated hepatic sinusoids.

#### 3.3.3. Titanium-Based Nanoparticles

**Figure 15:**
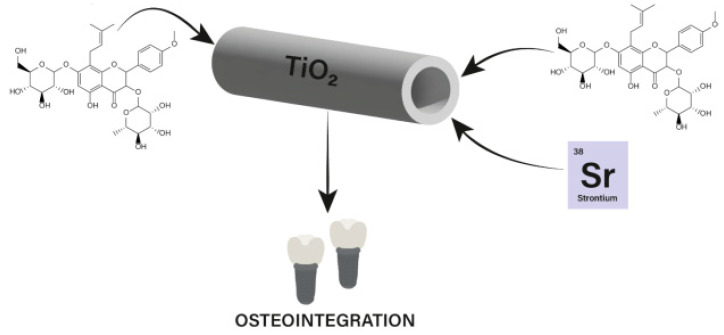
Strontium (Sr) and icariin-loaded TiO_2_ nanotube coatings to promote the osseointegration and early implant loading of titanium implants.

In the work of Zhu et al., the authors investigated the use of strontium, and ICA-loaded TiO_2_ nanotube coatings to promote the osseointegration and early implant loading of titanium implants in ovariectomized rats (Figure 15) [89]. The preparation included the formation of TiO_2_ nanotubes on titanium plates (Ti) by anodizing oxidation, consecutive thermal Sr coating, and the introduction of ICA onto the coating via chemical deposition. For analysis, the plates were separated into four groups. At different magnifications, SEM images of the surface morphology displayed that the Ti group had a smooth surface with some mechanical scrapes, the TiO_2_ group consisted of “honeycomb” nanotubes with ruptures on the surface, while TiO_2_ + Sr and TiO_2_ + Sr + ICA groups were irregular and different in size nanotubes with thickened walls and reduced tube holes. The EDS investigation confirmed the suggested composition for all groups. XRD investigation revealed the formation of pure Ti and anatase phases of TiO_2_ in all four groups, as well as the rutile phase of TiO_2_ in TiO_2_-bearing groups. In Sr-containing groups, the SrTiO_3_ was also detected. When compared to the Ti group, the TiO_2_-bearing groups showed higher surface roughness and hydrophilicity, and Sr^2+^ was released swiftly in the first week, followed by a constant release until day 30, whereas ICA had a burst release in the first 12 h and low thereafter. When compared to the Ti group, the TiO_2_-containing groups enhanced MC3T3-E1 cell adhesion, proliferation, alkaline phosphatase activity, mineralization, and osteogenic gene expression, with the TiO_2_ + Sr + ICA group having the highest results. In vivo tests back up these findings, revealing that the TiO_2_ + Sr + ICA group had the greatest bone-to-implant contact, bone density, and bone strength when compared to the other groups.

In their study from 2022, León-Gutiérrez et al. investigated the use of flavonoids, such as hesperetin 7-rutinoside (H7R) and flavanone-7-*O*-glucoside (F7G) adsorbed onto functionalized titanium dioxide nanoparticles (FTNP) as a potential antiviral against coronaviruses HCoV 229E and SARS-CoV-2 [90]. TiO_2_ nanoparticles (NPs) were prepared through adsorption and functionalized by adding various solutions containing functional groups to the NP suspension. The resulting functionalized NPs were characterized using XRD and TEM, which showed that they had an anatase structure with an average grain size of 2 nm. Organic extracts rich in H7R, F7G, and residual terpenes were then adsorbed onto the surface of the functionalized NPs through an impregnation process by stirring a suspension of the NPs in an aqueous solution of the extracts. Molecular docking studies were conducted to investigate how flavonoids or terpenes may interfere with the coupling between the SARS-CoV-2 spike protein and the human angiotensin-converting enzyme 2 (ACE-2) receptor. The study indicated that H7R and F7G had a high binding affinity to different sites on the spike protein with low-affinity energy values. Flavonoids were found to bind to specific regions of the spike protein, including the bottom and top of the S1 domain, near the binding site with ACE-2. The researchers hypothesized that these compounds might disrupt the interactions between the spike protein and ACE-2 or inhibit the necessary movement of the top region of the spike protein, potentially hindering the correct exposition of the binding site with ACE-2 and preventing COVID-19 infection. Molecular dynamics simulations were conducted under three conditions: free protein, protein/H7R, and protein/F7G, and lasted for 100 ns. An analysis of root mean square deviation (RMSD) and root mean square fluctuation (RMSF) showed that certain regions of the spike protein, including the amino and carboxyl-terminal regions and a loop in the middle section, had more movement than others. Cluster analysis was used to identify representative structures for each simulation, and the middle structures of these clusters were analyzed for protein–ligand interactions. The results showed that certain amino acids found in the docking analysis remained in close molecular interaction with the ligands throughout the simulations, constituting the binding site for H7R and F7G. These interactions included hydrogen bonds, with H7R forming more hydrogen bonds than F7G. The effects of FTNPs on the infectivity of CHoV-229E and SARS-CoV-2 were investigated in vitro. The incubation of CHoV-229E-infected MRC-5 cells and SARS-CoV-2-infected VERO.E6 cells with FTNP significantly reduced infection and viral replication. When FTNP was pre-incubated with SARS-CoV-2 before being added to VERO.E6 cells, there was a clear dose-dependent reduction in viral infectivity, while the pre-incubation with CHoV-229E could essentially eliminate any sign of infection in MRC-5 cells already under 1:2 dilution. FTNP also significantly increased the metabolic activity of SARS-CoV-2-infected cells, implying enhanced cell viability. To confirm the mechanism behind the protective effects of FTNPs against SARS-CoV-2 infection, the authors conducted a luciferase assay in co-transfected CHO-K1 cells and found that FTNPs significantly inhibited cell fusion. An increase in affinity between FTNPs and SARS-CoV-2 spikes was observed in dose-response studies, suggesting that FTNPs interfere with ACE-2 receptor-SARS-CoV-2 interactions.

Unnikrishnan et al. developed a method for synthesizing photocatalytic titanium oxide–gold nanocomposites (TiO_x_–Au NCs) with polycatechin shell [91]. To achieve these structures, HAuCl_4_, CT, and TiCl_3_ were combined at room temperature, framing target TiO_x_–Au nanocomposites (NCs). To make various NCs, the molar ratios of Ti^3+^ to Au^3+^ in the reaction mixture differed: 1:1, 5:1, 50:1, and 100:1. The resultant NCs were centrifuged and rinsed with ultrapure water. A similar procedure was used to create polycatechin-coated Au nanoparticles (Au@PC NPs) without the use of TiCl_3_ (the 0:1 reference). The generated nanoparticles displayed a variety of morphologies, ranging from a deformed sphere (TiO_x_–Au NCs_(1:1)_) to a sphere with tiny projections (TiO_x_–Au NCs_(5:1)_) or a star-like structure (TiO_x_–Au NCs_(50:1)_) and eventually to an urchin-like shape (TiO_x_–Au NCs_(100:1)_) when the Ti^3+^/Au^3+^ ratio increased. The light absorption characteristics of these TiO_x_–Au NCs altered considerably as the Ti^3+^ concentration increased. Au@PC NPs exhibited absorption at ~285 nm and ~410 nm, conditioned by the oxidized polycatechin shell, as well as the strong surface plasmon resonance (SPR) absorption at ~580 nm, which faded in favor of increased absorption at a longer wavelength with an increase in the Ti^3+^ presence. The TiO_x_–Au NCs series exhibited high light absorption in the NIR region due to a rise in the number of spikes with varying diameters and lengths as the Ti^3+^/Au^3+^ ratio increased. The photocatalytic capabilities of TiO_x_–Au NCs were investigated by evaluating the degradation of dyes methylene blue, rhodamine B (RhB), and malachite green under the illumination of a Xe Arc lamp. The dye degradation was examined between commercial TiO_2_ nanoparticles P25, Au@PC NPs, and all TiOx–Au NCs. TiOx–Au NCs had stronger photocatalytic activity than P25, most likely due to their localized surface plasmon resonance (LSPR) characteristics and polymer supramolecular “host–guest” chemistry (CT). The photocatalytic effectiveness rose for the series of NCs from TiOx–Au NCs (1:1) to TiOx–Au NCs_(50:1)_, and subsequently dropped for all three dyes at the greater Ti^3+^ concentrations. The broad range absorption of TiO_x_–Au NCs and localized surface plasmon resonance resulted in efficient hot electron production, which might provide more stable photogenerated electrons and holes for the breakdown of organic dye molecules. The inclusion of Au in the nanocomposite improved the conductance of electrons in the TiO_2_ conduction band to the surface, which facilitated electron transfer with dissolved O_2_ and dye molecules in the solution, advancing the degradation efficiency even more. TiO_x_–Au NCs_(50:1)_ were investigated further and shown to be effective for photocatalytic disinfection of both *E. coli* and MRSA bacteria in the presence of light. After 60 min of light exposure, the viability of these bacteria was decreased to less than 5% utilizing TiO_x_–Au NCs_(50:1)_, whereas P25 and the control revealed a minimal change in viability. The evidence of membrane shrinking and disruption of bacteria treated with TiO_x_–Au NCs_(50:1)_ was noted on TEM pictures after 30 min of light exposure. Intracellular ROS generation was also detected in bacteria treated with TiO_x_–Au NCs_(50:1)_ and subjected to light, but P25 and the control produced little ROS. The high levels of ROS produced during the photocatalytic process most likely caused oxidative stress in the bacteria, resulting in cell death. In the absence of light, the TiO_x_–Au NCs_(50:1)_ demonstrated antibacterial activity against MRSA, perhaps due to physical contact between the nanoparticles and the bacterial cell wall. The thin coating of polycatechin atop the TiO_x_–Au NCs_(50:1)_ may further contribute to antibacterial activity against Gram-positive bacteria via bacterial wall breakdown. Overall, the photocatalytic disinfection effectiveness of TiOx–Au NCs_(50:1)_ was good for both Gram-negative and Gram-positive bacteria. The PrestoBlue cell viability assay revealed that the cell viability of all four tested cell lines (human liver cancer HepG2, human embryonic kidney HEK-293 T, adenocarcinomic human alveolar basal epithelial A549, and mouse embryonic fibroblast NIH-3 T3) remained above 80%, even at 0.5 mg/mL of TiO_x_–Au NCs_(50:1)_, while sustaining the insignificant hemolytic effect. According to these findings, the nanomaterial is biocompatible with mammalian cells. TiO_x_–Au NCs_(50:1)_ were also reported to exhibit strong antibacterial activity against MRSA in a wound infection rat model. When compared to the control group and the group treated with P25 and light irradiation, the TiO_x_–Au NCs_(50:1)_-treated group with light irradiation demonstrated considerably quicker wound healing. Histological examination of infected tissues revealed that the TiO_x_–Au NCs_(50:1)_-treated group had much less inflammation and better-ordered tissue structure than the control group. These findings imply that TiO_x_–Au NCs_(50:1)_ have the potential to be a useful and effective antibacterial agent for wound healing. In the study published in 2022, Negrescu et al. explored the use of titanium dioxide nanotubes (TNT) loaded with icariin (ICA) through a layer of polydopamine (PDA) as a drug delivery platform for improved osseointegration in orthopedic implants [92]. As synthetic prerequisites for the fabrication of TiO_2_ nanotubes, titanium plates were prepared through a process of mechanical polishing and cleaning. The TNTs were then produced via anodic oxidation in an electrolyte solution and rinsed before being annealed. An adhesive intermediate layer of DP was applied to the nanotubes, followed by the immobilization of ICA through physical adsorption. The resulting samples were washed and used in further studies alongside flat titanium and unsheathed nanotubes. The characterization with SEM revealed that the resulting TNTs were found to be uniform and well-organized, with an inner diameter of around 65–70 nm and a wall thickness of 6–7 nm. When coated with DP, the tube diameter was reduced to 50–55 nm, and the thickness increased to 14–15 nm. AFM images of the uncoated and coated TNTs showed that the surface roughness increased from 54 nm for pure Ti to 95 nm for TiO_2_ nanotubes. After functionalization with a layer of DP and loading with ICA, the surface roughness increased to 159 nm, indicating that surface functionalization may alter the surface topography by elevating roughness and potentially influencing cell behavior. The hydrophilic nature and surface energy of unmodified and modified TiO_2_ nanotubes were evaluated using contact angle measurements. It was found that the surface energy increased from approximately 40 mJ/m^2^ for plain Ti to roughly 70 mJ/m^2^ for TNTs. The addition of a DP-ICA coating did not significantly affect the surface energy, indicating that the surface nanostructure has a greater impact on the final value than the coating. Electrochemical impedance spectroscopy (EIS) was used to assess the electrochemical behavior of the samples, revealing two different electrical circuits that were ascribed to the unmodified Ti sample and to the TNT and TNT-DP-Ica supports. The native TiO_2_ layer contributed highly to corrosion resistance, with values in the hundreds of kΩ for all three samples. Adding TiO_2_ in the form of nanotubes introduced a lower supplementary resistance of around 3 kΩ, due to the open nanotubular oxide structure. However, the addition of DP increased these supplementary values to 12 kΩ, possibly due to DP’s non-conductive nature. ICA was rapidly released from the TNT and TNT-DP samples in the first hour, with a rate of 10 μg/h and 6.5 μg/h, respectively. The release slowed significantly over the next 5 h, reaching rates of 5 μg/h for both samples. Over the next 168 h, the TNT support showed nearly no further ICA release, while the TNT-DP sample still presented a slow release rate of 0.04 μg/h. The impact of the tested surfaces on the survival rate of RAW 264.7 macrophages was examined using the Cell Counting Kit 8 assay and Live & Dead Cell Viability/Cytotoxicity Assay. The results showed that the coated TNT-DP-ICA sample had a lower number of viable metabolic active cells compared to the flat Ti and bare TNT substrates, but statistically, no significant differences were found. However, the TNT-DP-ICA sample had a lower cellular density and inhibited cell proliferation, while the bare TNT surface had no effect on cell survival or density. The effects of the tested surfaces on RAW 264.7 macrophages were investigated using Alex Flour 546-conjugated phalloidin in the actin cytoskeleton labeling. In the presence of *Escherichia coli* LPS, macrophages on flat Ti surfaces demonstrated increased spreading and an activated, migratory phenotype. However, TiO_2_ nanotubes protected against LPS-induced morphological changes, and the addition of ICA significantly reduced these changes. Quantitative analysis also showed that coated TNT surfaces inhibited the activation of macrophages. The secretion of pro-inflammatory mediators such as IL-6, TNF-α, and MCP-1 by RAW 264.7 cells was measured after 24 h of culture using an ELISA. The results showed that the levels of these cytokines increased significantly in the presence of LPS, with the exception of IL-6, which was only detectable after LPS stimulation. The levels of IL-6 and MCP-1 were lower in cells grown on the TNT-DP-ICA surface compared to the flat Ti and bare TNT surfaces, while the levels of TNF-α were similar across all samples. The ability of the samples to stimulate the release of NO by macrophages in the presence of LPS was also analyzed by measuring the levels of nitrite in the culture media. In standard culture conditions, the levels of NO were low and comparable across all samples. However, in the presence of LPS, the cells grown on the flat Ti surface released the highest levels of NO, while the TNT-DP-ICA surface elicited the lowest levels of NO compared to the flat Ti and bare TNT surfaces. In an in vivo study using rats, the effects of various titanium implants on bone formation were assessed. The rats received implants in their posterior legs; some received unmodified Ti implants, others received Ti implants with a layer of TiO_2_ nanotubes, and others received Ti implants with a layer of TiO_2_ nanotubes and ICA functionalization through an intermediate DP layer. X-ray and histological analyses of the harvested bone tissue were performed at 1- and 90-days post-implantation. The results showed that the Ti implants with TiO_2_ nanotubes and ICA functionalization revealed the most pronounced bone formation, while the unmodified Ti implants had minimal bone formation and dense fibrous tissue. Inflammation was not observed in any of the implants.

#### 3.3.4. Ceria-Based Nanoparticles

Thakur et al. studied the usage of amine-functionalized ceria nanoparticles (CeO_2_-NH_2_NPs) as a medication delivery vehicle for a natural flavonoid morin against both Gram-negative and Gram-positive bacteria [93]. CeO_2_NPs were made by combining ammonium cerium nitrate, urea, and sodium hydroxide in water and refluxing it for 5 h. After that, the NPs were functionalized with 3-aminopropyl triethoxysilane (APTES) by combining them with APTES and refluxing the mixture for 12 h. Morin-CeO_2_-NH_2_NPs were made by combining morin with a dispersed solution of amine-functionalized NPs in ethanol and swirling the mixture continuously for 12 h. CeO_2_NPs with a size of 3–4 nm were characterized using TEM, XPS, and XRD. The nanoparticles were found to have a cubic fluorite structure and were surface functionalized with APTES, as confirmed by FTIR. The hydrodynamic size of the nanoparticles was determined to be 86.11 nm using DLS, and their zeta potential was found to be +23.2 mV. The UV-visible spectra of the nanoparticles displayed strong absorption in the 300–325 nm range. Morin was also incorporated into the nanoparticles, resulting in an absorption band in the 300–400 nm range as determined by UV-vis spectroscopy. Morin drug loading content on the nanoceria surface was around 10%. Morin was released from the nanohybrid in a time-dependent manner, with 30.65%, 43.79%, 45.98%, and 54.74% released after 6, 12, 24, and 48 h, respectively. The nanohybrid demonstrated concentration-dependent radical scavenging activity in DPPH, hydrogen peroxide, and superoxide radical scavenging experiments, with maximal scavenging of 50%, 87%, and 70%, respectively. When normal kidney epithelial (NKE) cells were treated with the nanohybrid before being exposed to tertiary butyl hydroperoxide, an ROS inducer, the nanohybrid was observed to lower intracellular ROS levels when compared to free morin or CeO_2_-NH_2_NPs. The nanohybrid triggered dose-dependent cytotoxicity in NKE cells, with cell viability reaching 80.67% at 50 μg/mL and surpassing 65% at 100 μg/mL. Morin-CeO_2_-NH_2_NPs inhibited growth in both Gram-positive (*S. aureus*) and Gram-negative (*E. coli*) bacteria and exhibited the highest growth inhibitory efficacy among the formulations evaluated. Their MIC values against the studied microorganisms were 120.75 μg/mL for *S. aureus* and 165.28 μg/mL for *E. coli*, respectively. Among the investigated formulations, the nanohybrid produced the highest DNA degradation in both S. aureus and *E. coli* bacteria, and the degree of degradation was greater in *S. aureus*. It raised ROS levels in *S. aureus* and *E. coli* bacteria by 4- and 5.5-fold, respectively, compared to control groups, and increased membrane permeability, depolarization, and cell shrinkage, while elevating LDH release in both types of bacteria. Overall, the results demonstrate that morin-CeO_2_-NH_2_NPs have strong antioxidant and antibacterial capabilities, with low toxicity toward NKE cells at 50 μg/mL concentration.
**To sum up****Pros**Connections of flavonoids with metal oxide nanoparticles, mainly zinc oxide, ce-ria, titanium-based, CuO/ZnO, and titanium oxide–gold nanocomposites, resulted in materials of interesting physicochemical and biological properties, including antibacterial, antifungal, antiviral, anticancer, and prospective applications as drug delivery platforms for improved osseointegration in orthopedic implants.Metal oxide nanoparticles, such as zinc oxide, ceria, titanium-based, CuO/ZnO, and titanium oxide–gold, were functionalized with quercetin, EGCG, baicalin, lu-teolin, chrysin, morin, icariin hesperetin 7-rutinoside, flavanone-7-O-glucoside to study their prospective medical applications.The ability of the UV light absorption of zinc oxide nanoparticles indicates their perspective application in antiphotoaging therapy.**Cons**The effects of ZnO-NPs that could be safely applied in the biomedical field have been intensively studied to understand the interplay between their physicochem-ical properties and toxic effects. The safety and toxicity issues of ZnO-NPs have become a field of increased public attention [94,95].Titanium dioxide is a commonly used food, cosmetic, and drug additive or ingre-dient. Everyday use of nanosize TiO2 raises concerns about safety. There are vari-ous data demonstrating the toxic effects of titania in animal models. An agree-ment on the safety of titania has not yet been reached among researchers [96].

CuO/ZnO NCs functionalized with quercetin presented biocidal effectiveness [85]. ZnO nanosystems combined with EGCG, CHI, and PLGA were found to prevent UVB-induced skin damage [88], and quercetin-loaded ZnO NPs show promising potential for use in antiphotoaging therapeutics [86]. Although ZnO NPs are often applied as nanocarriers in anticancer procedures such as the ZnO-PBA (phenylboronic acid) to lung cancer cells [84] or for action potentiation of active flavonoid such as ZnO@Quercetin towards human ovarian cancer [83], they can also be combined with luteolin to improve insulin resistance and reduce the activity of liver enzymes [87]. Apart from iron or zinc oxide NPs, nanoforms of titanium dioxide are often investigated. A drug delivery platform consisting of titanium dioxide nanotubes loaded with ICA through a layer of PDA contributed to improved osseointegration in orthopedic implants [92], whereas flavonoid compounds adsorbed on FTNP revealed antiviral potential against coronaviruses HCoV 229E and SARS-CoV-2 [90]. The summary of the data related to connections of selected metal oxide nanoparticles with flavonoids, which were presented in the section, was included in Table 4.

## 4. Summary and Conclusions

Broad interest in hybrid materials based on metal nanoparticles and flavonoids has increased due to their unique physical, chemical, and biological properties. The widespread applications of nanomaterials based on flavonoids and metal nanoparticles constitute a promising perspective for both interesting basic study and practical applications.

**Figure 16:**
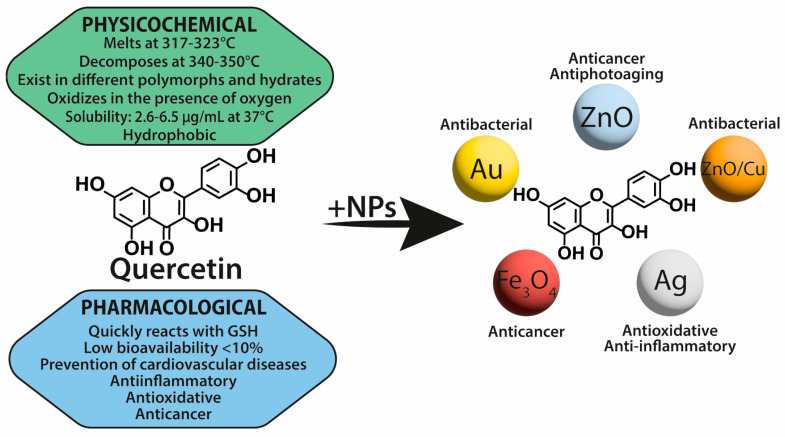
Quercetin and its physicochemical and pharmacological [97,98,99] properties as well as prospective biological and medical activities in connections with nanoparticles.

Flavonoid-modified metal nanoparticles demonstrate their utilities from the initial preparation step to advanced medical and pharmaceutical studies. The review combines knowledge of multiple possibilities of synthesis of flavonoid-metal nanoparticle conjugates and hybrids via their characterization, biological properties, and medical applications. Many research results have led to the conclusion that the combination of flavonoids with metal nanoparticles results in the improvement of their individual properties and applications. The combinations of the flavonoid quercetin with various nanoparticles discussed in this review can be used to indicate the effects caused by the nanoparticle on the flavonoid molecule, and vice versa. The obtained nanoparticles functionalized with flavonoids have not only been endowed with modified physicochemical properties, but their biological activity has also been extended, and thus the prospects for their applications in medical sciences (Figure 16).

Silver nanoparticles conjugated with flavonoids exerted satisfying antibacterial, anti-inflammatory, antioxidant, anticancer, and antifungal activities, as well as diagnostic applications. Moreover, silver nanoparticles modified with zinc or gold revealed antiviral and antiparasitic activity potential. Functionalizing gold nanoparticles with flavonoids improved their properties and broadened the spectrum of applications. Gold nanorods with metal-phenolic networks used with NIR-irradiation significantly accelerated the healing of infected wounds and proved their bactericidal effectiveness. Selected flavonoid-conjugated nano-gold particles exerted antibacterial, antiparasitic, antiangiogenic, anticancer, and antioxidant activity, as well as being studied for diagnostic applications. Iron oxide nanoparticles were produced and formulated with flavonoids for applications in the treatment of various cancers. They were also applied as components of drug delivery systems for multimodal synergistic cancer therapy. Zinc oxide nanosystems combined with selected flavonoids were found to prevent UVB-induced skin damage and demonstrated promising potential for use in antiphotoaging therapeutics. They were also proposed as nanocarriers in anticancer procedures and metabolic studies. Titanium dioxide connections with flavonoids were studied against viruses and proposed as a drug delivery platform of the greatest bone-to-implant contact allowing improved osseointegration in orthopedic implants.

To sum up, flavonoid-metal nanoparticles have potential as agents for the treatment of various diseases due to anti-inflammatory, bactericidal, antifungal, antiviral, or antiparasitic properties, as well as for the preparation of modern DDSs and diagnostic agents for computed tomography and magnetic resonance imaging. Although novel nanoparticles offer many advantages to health therapies, some of their properties, such as biodegradability and elimination from the human organism, raise objections and still require more research.

## Figures and Tables

**Table 1:** The summary of the data presented in Section 3.1.

Flavonoid/ Compound	Size of NPs	Synthesis Method	Activity	Target	Ref.
Hesperidin, Pectin	11.93–17.34 nm	microwave-assisted reduction	MIC 66.7 μg/mL	*E. coli*	[43]
Hesperidin	~20 nm	chemical reduction of AgNO_3_	inhibition rate of 94.5%	*S. aureus*	[44]
Kaempferol, Hydrocortisone	10–30 nm	chemical reduction of AgNO_3_ with NaBH_4_	MIC 62.5 μg/mL	*E. coli*	[45]
Catechin Myricetin	5 nm (AgNPs@MY) and 8 nm (AgNPs@CT)	chemical reduction of AgNO_3_ in alkaline environment	33% of growth inhibition at conc. 30 mg/L of AgNPs@CT	*Aspergillus niger*	[46]
EGCG	Not specified	chemical reduction of AgNO_3_ with NaBH_4_	7.1 reduction in the logEID_50_/mL value	H5N1 influenza	[47]
4′,7-Dihydroxyflavone	25.1 nm	chemical reduction of AgNO_3_	0.8483 µg/mL 0.262 µg/mL	promastigotes amastigotes	[48]
Apigenin	93.94 nm	chemical reduction of AgNO_3_	MIC 0.06–3.75 mg/mL	Gram-positive and Gram-negative bacteria	[49]
Myricetin	12–20 nm (TEM)	microwave-assisted reduction and aging	IC_50_ 34.04 μg/mL	Human colorectal cancer cells (HCT116)	[50]
Dihydromyricetin	114.76 nm	solvothermal method	MIC 10^−6^ g/L (*E. coli*) and 10^−4^ g/L (*Salmonella*) Cell viability = approx. 18.5%	*E. coli* and *Salmonella* MDA-MB-231 breast cancer cells	[51]
Quercetin	35 nm	chemical reduction of AgNO_3_	Cancer cells imaging	HeLa cells	[52]
Curcumin, Quercetin	32.71 nm (hydrodynamic size)	chemical reduction of AgNO_3_	Anti-inflammatory activity	Mouse macrophages	[53]
Isoorientin	117 nm	green synthesis approach from corn starch	Not specified	HL-7702 human liver cells	[54]

**Table 2:** The summary of the data presented in Section 3.2.

Flavonoid/ Compound	Size of NPs	Synthesis Method	MIC/ID_50_	Action	Ref.
4′,7-Dihydroxyflavone	8 nm	chemical reduction of HAuCl_4_	IC_50prom_ 0.1226 µg/mL IC_50ama_ 0.115 µg/mL	promastigotes amastigotes	[48]
Chrysin, kaempferol, and quercetin	4.1–35 nm	chemical reduction of HAuCl_4_ with NaBH_4_ in the presence of glutathione	MIC 30 μg/mL for quercetin-AuNPs	*E. coli*, *P. aeruginosa*, and *P. vulgaris*	[58]
EGCG, procyanidins, tannic acid	Not specified	seed-mediated growth method	98.6% *S. aureus* growth suppresion and 99.8% *E. coli* growth suppression for procyanidine-AuNPs	*S. aureus* and *E. coli*	[59]
Quercetin	30 nm (HRTEM)	reduction of HAuCl_4_ by sonication	MIC 7.6 μg/mL	*E. coli*	[62]
Chrysin	20 nm	chemical reduction of HAuCl_4_ in the acidic pH and high temperature	IC_50_ 0.8 μg/mL	*L. donovani*	[63]
EGCG	125 ± 13 nm	chemical reduction of HAuCl_4_ following with carbodiimide-mediated cross-linking of EGCG	50% cell growth inhibition at EGCG concentrations of 2.2 μM	Pancreatic cancer cells	[64]
EGCG, ECG	Not specified	chemical reduction of HAuCl_4_	Not specified	*E. coli*	[65]
Catechin modified with linker	1.7 nm	chemical reduction of HAuCl_4_ in the presence of glutathione	Not applicable	Fluorescent nanoprobe for detection of dopamine	[66]
Hesperidin	18–32 nm	chemical reduction method with trisodium citrate	degradation rate > 90% (methyl orange, methylene blue, bromocresol green, 4-nitrophenol); IC_50_ 37.16 μg/mL	Photocatalyst for the treatment of industrial wastewater; antioxidant activity	[67]
Catechin	Ag/Au NPs Not specified	chemical reduction of HAuCl_4_ and AgNO_3_ by NaOH	detection limit of 0.4 μM	glucose detection	[68]

**Table 3:** The summary of the data presented in Section 3.3.1.

Flavonoid Type	Type of NPs	Size of NPs	Synthesis Method	MIC/ID_50_	Action	Ref.
Curcumin, silymarin	SPIONs	57 nm	reverse microemulsion method	Not specified	MCF-7 cells	[11]
Quercetin	Fe_3_O_4_	40 nm	enzymatic reduction of FeSO_4_	Cell viability ≤ 9%	MCF-7 cells	[73]
Quercetin	Fe_2_O_3_	148.2 nm	chemical reduction of Fe(NO_3_)_3_·9H_2_O by NH_3_·H_2_O	Not specified	MCF-7 cancer cells	[74]
Quercetin	Fe_3_O_4_	8.7 nm	co-precipitation in an aqueous solution	Not specified	Human ovarian carcinoma cells	[75]
Eupatorin	Fe_3_O_4_	58.5 nm	nano-precipitation approach	IC_50_ 100 mM and 75 mM	Human prostate cancer cell lines DU-145 and LNCaP	[76]
Silibinin	Fe_3_O_4_	285.9 nm	co-precipitation of FeCl_2_ in the presence of H_2_O_2_	IC_50_ 3 μg/mL	A-498 human kidney cancer cells	[77]
EGCG, epicatechin, proanthocyanidin	Fe	21 nm for Fe-EGCG, 27 nm for Fe-EC, and 30 nm for Fe-PAC	sol-gel process	Not specified	HeLa cells	[78]
EGCG	Fe_3_O_4_	150–180 nm	co-precipitation of FeCl_2_ and FeCl_3_ with NH_4_OH	Cell viability ≤ 10%	B16F10 cells	[79]
Quercetin	Fe_3_O_4_	10–15 nm	co-precipitation method	non-toxic for isolated rat mitochondria	multifunctional drug delivery platform	[80]
Baicalin	Fe_3_O_4_	100 nm	Commercially available Fe_3_O_4_@SiO_2_ nanoparticles were used	protein capture efficiency of 19.69 µg/mg	Human embryonic kidney cells (HEK293)	[81]

**Table 4:** The summary of the data presented in Section 3.3.2, Section 3.3.3 and Section 3.3.4.

Flavonoid Type	Type of NPs	Size of NPs	Synthesis Method	MIC/ID_50_	Action	Ref.
Luteolin	ZnO	17 nm	chemical reduction of zinc acetate	Cell viability = 15% at 40 μM	MCF-7 cells	[82]
Quercetin	ZnO	12–18 nm	chemical reduction of zinc nitrate by KOH	IC_50_ = 10 μg/mL	human ovarian cancer cells	[83]
Chrysin	ZnO	25–30 nm	chemical reduction of zinc acetate	cytotoxicity observed in the A549 cells at 16.3 μg/mL	A549 and L132 lung cancer cell lines	[84]
Quercetin	CuO/ZnO	Not specified	co-precipitation of copper acetate, zinc acetate and quercetin; calcination	Zone inhibition method (in mm) for conc. 500 μg/mL	*E. coli*, *S. aureus*, *Shigella,* and *Bacillus subtilis*; *Aspergillus niger* and *Candida albicans*	[85]
Quercetin	ZnO	Not specified	chemical reduction of zinc acetate	Cell viability = 80% after 24 h	antiphotoaging therapy HaCaT cells	[86]
EGCG	ZnO	152 nm	co-precipitation method	20–45% reduction of UVB-generated CPDs and 20–48% reduction of 6-4PPs	UVB-exposed SKH-1 hairless mice (chemopreventive agent)	[88]
Luteolin	ZnO	17 nm (TEM)	chemical reduction of zinc acetate by sodium hydroxide	Not applicable	decrease in the insulin resistance	[87]
Icariin	TiO_2_	50 nm diameter	anodizing oxidation	Not applicable	drug delivery platform for improved osseointegration	[89]
Hesperetin 7-rutinoside (H7R) and flavanone-7-O-glucoside (F7G)	TiO_2_	2 nm	adsorption process	dose-dependent reduction in viral infectivity	antiviral agents against coronaviruses HCoV 229E and SARS-CoV-2	[90]
Catechin	TiOx–Au NCs	144.3–338.9 nm (hydrodynamic sizes depending on core composition)	Reaction of HAuCl_4_, catechin and TiCl_3_ at rt	Cell viability ≤ 5%	*E. coli* and MRSA	[91]
Icariin	TiO_2_	50–55 nm × 14–15 nm	mechanical polishing and cleaning process	Not applicable	drug delivery platform for improved osseointegration	[92]
Morin	CeO_2_	86.11 nm	chemical reduction of cerium nitrate by NaOH in the presence of urea	MIC = 120.75 μg/mL for *S. aureus* and 165.28 μg/mL for *E. coli*	*S. aureus* *E. coli*	[93]

## Data Availability

Not applicable.

## Abbreviations

47DHF, 4′,7-dihydroxyflavone; 4NP, 4-nitrophenol; Ag@QCNPs, silver@quercetin nanoparticles; Ag-Hes NP, hesperidin-capped silver nanoparticle; AgNPs-Iso, isoorientin-loaded silver nanoparticles; AIEgen, aggregation-induced emission luminogen; AP, apigenin; BE, phenylboronic acid; C-Au NC, catechin-functionalized gold nanocluster; CC_50_, 50% cytotoxic concentration; CeO_2_-NH_2_NP, amine-functionalized ceria nanoparticle; CHI, chitosan; CHY, chrysin; CHY-AuNPs, chrysin-gold nanoparticles; CMC, carboxymethylcellulose; CT, catechin; CTAB, cetyltrimethylammonium bromide; DA, dopamine; DMY, dihydromyricetin; DPPH, 2,2-diphenyl-1-picrylhydrazyl; EC, epicatechin; ECG, (−)-epicatechin gallate; EDS, energy dispersive spectroscopy; EDX, energy-dispersive X-ray; EGCG, epigallocatechin gallate; F7G, flavanone-7-*O*-glucoside; FBS, fetal bovine serum; FESEM, emission scanning electron microscopy; GA, gallic acid; GNP, gold nanoparticle; GNR, gold nanorods; GOx, glucose oxidase; GSH, glutathione; GTP, green tea polyphenols; H7R, hesperetin 7-*O*-rutinoside; Hes, hesperidin; HP-AgNPs, silver nanoparticles coated with hesperidin and pectin; ICA, icariin; ICG, indocyanine green; IO, iron oxide; Iso, isoorientin; KH-AgNPs, silver nanoparticles conjugated with kaempferol and hydrocortisone; LDH, lactate dehydrogenase; LPS, lipopolysaccharide; LSPR, localized surface plasmon resonance; Lut, luteolin; MDA, malondialdehyde; MIC, minimum inhibitory concentration; MNPs, iron oxide nanoparticles; mPEG, methoxy poly(ethylene glycol); MY, mycerin; OPC, procyanidin; PAC, proanthocyanidin; PC, phenolic compound; PCL, poly(ε-caprolactone); PDA, polydopamine; PI, propidium iodine; PVA-Alg, poly(vinyl alcohol)-sodium alginate; QMNPs, quercetin-conjugated magnetite nanoparticles; QUR, quercetin; RSC, radical scavenging capacity; RSV, resveratrol; SAED, selected area electron diffraction; SC-SNPs, sodium citrate-based silver nanoparticles; SI, selectivity index; SIL, silymarin; SLB, silibinin; SLB-MPNP, PLGA-encapsulated silibinin; SPION, super-paramagnetic iron oxide nanoparticle; SPR, surface plasmon resonance; TA, tannic acid; TEOS, tetraethyl orthosilicate; TiOx–Au NC, titanium oxide-gold nanocomposite; TNT, titanium dioxide nanotubes.
